# Association between neuroticism and brain-wide structural outcomes: Mediation by vascular and mental conditions

**DOI:** 10.1017/S0033291725102390

**Published:** 2025-11-14

**Authors:** Yaqing Gao, Bernd Taschler, Najaf Amin, Cornelia van Duijn, David J. Hunter, Anya Topiwala, Thomas J. Littlejohns

**Affiliations:** 1 Nuffield Department of Population Health, University of Oxford, Oxford, UK; 2 Big Data Institute, Nuffield Department of Medicine, University of Oxford, Oxford, UK; 3 Department of Epidemiology, Harvard TH Chan School of Public Health, Boston, MA, USA; 4 Big Data Institute, Nuffield Department of Population Health, University of Oxford, Oxford, UK

**Keywords:** genetics, imaging, MRI, neuroticism, personality

## Abstract

**Background:**

Neuroticism, a personality trait linked to both cardiovascular and psychiatric disorders, has been associated with cognitive decline and increased dementia risk, though the underlying neural mechanisms remain unclear. Mapping its relationship with brain structure could provide valuable insights into neural pathways and targets for early intervention.

**Methods:**

We examined brain-wide associations between neuroticism and structural neuroimaging metrics derived from T1-, T2-weighted, and diffusion MRI in 36,901 dementia-free UK Biobank participants. Bonferroni-significant associations underwent bidirectional two-sample Mendelian randomization to evaluate the evidence for a causal relationship. Given that neuroticism is generally stable across adulthood and challenging to modify, we assessed whether these associations were mediated by health conditions (depression, anxiety, hypertension, ischemic heart disease [IHD], and diabetes) that are both consequences of neuroticism and known risk factors for dementia, and also modifiable through widely available and efficacious therapeutic interventions.

**Results:**

Higher neuroticism was found to be associated with reduced grey matter volumes in the frontal and limbic regions, as well as widespread differences in white matter microstructure, particularly in thalamic radiations. Genetic analyses supported a potential causal effect of neuroticism on increased diffusivity in thalamic radiations. Hypertension mediated the associations between neuroticism and both grey and white matter measures, while depression and anxiety primarily mediated associations with white matter microstructure. Contributions from IHD and diabetes were minimal.

**Conclusions:**

Neuroticism is linked to widespread structural brain differences that contribute to poorer brain health, and targeting vascular and mental health may help mitigate its impact.

## Introduction

Neuroticism, a stable and common personality trait marked by irritability, worry, mood swings, and other negative emotions, is a major public health concern linked to a wide range of physical and psychiatric health outcomes (Widiger & Oltmanns, 2017), including poorer cognitive function and increased dementia risk (Aschwanden et al., 2021; Beck et al., 2024; Gao, Amin, van Duijn, & Littlejohns, 2024; Terracciano et al., 2021). However, the neural mechanisms underlying this link remain unclear. Previous studies have identified associations of neuroticism with reduced global grey matter volume and increased white matter hyperintensities (WMH) (Chen & Canli, 2022; Gao et al., 2024; Terracciano et al., 2023), suggesting a broad neurodegenerative and cerebrovascular burden. To better understand the neurobiological pathways involved, it is essential to examine neuroticism in relation to specific brain structures, such as regional grey matter volumes and the microstructure of individual white matter tracts. Identifying such early, subtle structural differences, particularly before clinical cognitive decline, may inform targeted strategies for early dementia prevention.

Evidence linking neuroticism to specific brain structures remains limited. Prior research has largely focused on grey matter volume. A recent meta-analysis of 17 whole-brain studies (mean age 34 years; most with sample sizes under 100) found no significant associations between neuroticism and regional cortical or subcortical volumes (Chen & Canli, 2022), despite some individual studies reporting smaller frontal lobe volumes (DeYoung et al., 2010; Kapogiannis, Sutin, Davatzikos, Costa, & Resnick, 2013; Lu et al., 2014; Owens et al., 2019). Even less evidence exists for white matter microstructure, which can be assessed using diffusion tensor imaging (DTI) and serves as an early and sensitive indicator of vascular-related brain changes (Iadecola, 2013; Power et al., 2017). DTI studies in adolescent cohorts reported null associations (Avinun, Israel, Knodt, & Hariri, 2020; Bjørnebekk et al., 2013; Delaparte et al., 2019), but three adult studies (*N* = 51, mean age 30 years (Xu & Potenza, 2012); *N* = 668, mean age 73 years (McIntosh et al., 2013); and *N* = 265, mean age 50 years (Bjørnebekk et al., 2013) consistently found neuroticism-related reductions in fractional anisotropy (FA) and increases in mean diffusivity (MD) across multiple white matter tracts.

These initial findings have several key limitations. First, most studies have been underpowered, with sample sizes below 100. A minimum sample size of 1,000 has been recommended to reliably detect associations between brain structure and psychological traits, including personality (Kharabian Masouleh et al., 2019; Marek et al., 2022). Second, most samples were composed of younger adults, making examination of age- or dementia-related structural differences impossible. Third, previous research has neglected other neuroimaging modalities. For example, neurite orientation dispersion and density imaging (NODDI) offers greater specificity for features such as axonal density and fiber orientation and thus biological insights (Zhang, Schneider, Wheeler-Kingshott, & Alexander, 2012), yet its association with neuroticism remains unexamined. Additionally, neuroticism has not been explored in relation to other emerging preclinical markers of dementia, such as ventricular enlargement (Nestor et al., 2008) and reduced cerebellar volume (Guo et al., 2016).

A crucial unanswered question is whether neuroticism leads to structural brain changes, or conversely, whether brain structure influences neuroticism tendencies. Cross-sectional observational studies conducted to date have been unable to distinguish between these two possibilities. However, analyzing associations with genetic variants inherited at meiosis, Mendelian randomization analysis offers a powerful method to address this issue, as it can help establish the directionality of the relationship between neuroticism and brain structure.

Given the relative stability of personality traits across adulthood (Bleidorn et al., 2022), direct and sustainable modification of neuroticism remains challenging (Stieger et al., 2021). However, targeting modifiable downstream consequences of neuroticism may offer a practical approach to mitigate its potential impact on brain health. We focused on mental and vascular conditions, which are not only consequences of neuroticism (with established causal links) (Howard et al., 2019; Nagel et al., 2018; Zhang et al., 2021) and risk factors for dementia (Livingston et al., 2024), but also have widely available interventions. Prior research suggests that the link between neuroticism and dementia risk is likely driven by these conditions (Gao et al., 2024). However, their role in neuroticism–brain structure associations remains unexplored.

UK Biobank, a large population-based cohort of middle- to older-aged adults with multimodal brain imaging and extensive sociodemographic and health-related data (Sudlow et al., 2015), is uniquely suited for brain-wide neuroimaging analyses and pathway exploration. A recent UK Biobank study found cortical and white matter differences—previously linked to brain disorders—associated with proxy measures of personality traits (Zhang et al., 2023), but relied on non-validated proxies and a limited set of imaging outcomes. Leveraging the large UK Biobank sample, we used a validated neuroticism scale to examine associations across a comprehensive range of imaging metrics, including global measures, regional cortical and subcortical volumes, surface area, thickness, signal intensity, white matter microstructure (traditional and NODDI-derived), and ventricular and brainstem volumes. We further assessed whether associations were mediated by modifiable health conditions linked to neuroticism or dementia (depression, anxiety, and stress-related disorders, ischemic heart disease [IHD], hypertension, and diabetes). Additionally, we applied the first bidirectional two-sample Mendelian randomization (MR), a quasi-experimental approach that uses genetic variants as instruments, to investigate potential causal effects between neuroticism and brain structure.

## Methods

### Study participants

The UK Biobank is a prospective cohort study that recruited over 500,000 participants aged 40 to 69 across the United Kingdom between 2006 and 2010 (Sudlow et al., 2015). In 2014, UK Biobank launched its imaging study, inviting participants to undergo multimodal brain MRI scans (Littlejohns et al., 2020). The scans were performed at three centers (Newcastle upon Tyne, Stockport, and Reading) using 3 Tesla Siemens Skyra scanners (Siemens Healthineers) equipped with VD13 software and 32-channel head coils, following standardized operating procedures (Miller et al., 2016).

During the imaging visit, participants also provided comprehensive demographic, socioeconomic, lifestyle, and medical information through self-administered touchscreen questionnaires and verbal interviews conducted by trained nurses. National inpatient records for all participants (Hospital Episode Statistics [HES] for England, the Patient Episode Database for Wales, Public Health Scotland) and death registration records were linked to the UK Biobank.

We included participants with valid measurements for at least one neuroimaging outcome of interest as of June 30, 2024 (*n* = 47,503). Participants were excluded if they self-reported a diagnosis of dementia/cognitive impairment or had a dementia diagnosis in inpatient records (*n* = 30), or had missing data for neuroticism (*n* = 9,156). Supplementary Table S1 provides a comparison of characteristics between participants with and without missing neuroticism data. Due to the low proportion of missing covariate data (2.9% for BMI and <1% for other covariates; Supplementary Table S1), we further excluded participants with missing covariate data (*n* = 1,422).

### Assessment of neuroticism

We used the neuroticism measurement obtained at the imaging visit, which was self-reported through the touchscreen questionnaire using the 12-item Eysenck Personality Questionnaire-Revised Short Form (EPQ-RS) (Supplementary Table S2) (Eysenck, Eysenck, & Barrett, 1985). Each item assessed the presence of a neurotic trait with a binary response (‘Yes’ = 1, ‘No’ = 0). The total neuroticism score was calculated as the sum of all item scores, ranging from 0 to 12, with higher scores indicating greater neuroticism. The EPQ-RS has shown high reliability (Cronbach’s alpha >0.8) (Eysenck et al., 1985) and strong correlations (*r* = 0.85) (Gow, Whiteman, Pattie, & Deary, 2005) with other established neuroticism measures. To enable direct comparisons across studies with different neuroticism measures and health outcomes, we standardized neuroticism scores to a mean of 0 and a standard deviation (SD) of 1 (z-score) (Aschwanden et al., 2021).

### IDP selection

Brain structure measurements were obtained from three imaging modalities: T1-weighted MRI, T2-weighted fluid-attenuated inversion recovery (FLAIR), and diffusion MRI. T1-weighted imaging provided global and regional volume estimates for a wide range of brain structures, T2-FLAIR identified white matter hyperintensities, and diffusion MRI assessed white matter microstructure. The imaging data from these modalities were processed, quality-controlled, and released as imaging-derived phenotypes (IDPs) by the UK Biobank team (Miller et al., 2016).

For this study, we focused on structural IDPs, including measures of global and regional cortical volumes, surface area, thickness, and intensity; cerebrospinal fluid (CSF) volumes; subcortical structure volumes and intensity; white matter hypo- and hyperintensities; and white matter microstructure. Among the available IDPs, we excluded those that had been pre-adjusted for head size by the UK Biobank, as we applied our own adjustments for imaging confounds. Additionally, we generated new IDPs for radial diffusivity in white matter tracts by averaging the diffusivity parameters (λ2 and λ3), while removing the original λ2 and λ3 IDPs. Ultimately, 1,747 IDPs were included for analysis. Detailed processing pipelines are described in the Supplementary Methods, and the complete list of IDPs is included in Supplementary Table S3.

To enhance the robustness of association estimates, we applied a rank-based inverse Gaussian transformation (quantile normalization) to all IDPs (Miller et al., 2016). A total of seven image-related confounds were adjusted in our analysis, including head size (based on the volumetric scaling from the T1 head image to standard atlas), head motion (mean absolute head motion from diffusion MRI calculated by Eddy), head position (X, Y, Z brain center of gravity, and table position), and imaging center (Supplementary Table S4) (Alfaro-Almagro et al., 2021). These image-related confounds were included as predictors in a linear regression model with IDPs as the dependent variables. The residuals from these models were then standardized for use in further analyses.

### Assessment of covariates and mediators

We adjusted models for the following covariates: sex, age (continuous), polynomial terms for age (age^2^ and age^3^) to account for nonlinear age effects across outcomes (Alfaro-Almagro et al., 2021), age × sex to capture sex-specific patterns (Alfaro-Almagro et al., 2021), self-reported ethnicity (white and non-white; using the first non-missing value across all assessments), quintiles of the Townsend deprivation index (computed from national census data preceding baseline and assigned based on the participant’s post-code), education (primary, secondary, post-secondary non-tertiary, and tertiary), smoking status (never, previous, current), drinking (≤ 4 times per week, daily or almost daily), and body mass index (BMI; <25 [normal], 25–29.9 [overweight], ≥ 30 [obese]). All covariates, except ethnicity and Townsend deprivation index, were measured at the imaging visit. Supplementary Table S4 provides detailed information on the measurement, definition, and classification of the covariates.

To ensure the temporal sequence of neuroticism preceding mediators, we selected health conditions identified in Mendelian randomization studies as influenced by neuroticism, rather than the reverse. Specifically, we identified mental conditions (depression, anxiety, and stress-related disorders) and vascular conditions (IHD, hypertension) as mediators, as neuroticism increased their risk without these conditions significantly impacting neuroticism levels (Howard et al., 2019; Nagel et al., 2018; Zhang et al., 2021). Additionally, we included diabetes as a negative control mediator, given its established association with poor brain health but limited evidence of an association with neuroticism (Antal et al., 2022; Zhang et al., 2021). We anticipated that the mediation effect of diabetes would be smaller or non-significant compared to the mental and vascular conditions. These conditions were ascertained through verbal interview and linked hospital inpatient records preceding the imaging visit, with diagnostic codes detailed in Supplementary Table S4.

### Genetic variants

Genetic associations with neuroticism and IDPs were derived from the largest publicly available genome-wide association studies (GWAS) of European ancestry. The GWAS for neuroticism included 240,000 individuals of European ancestry from the Million Veteran Program cohort, with summary statistics meta-analyzed with data from the UK Biobank, 23andMe, and GPC phase 1, resulting in a total sample size of 682,688 (Gupta et al., 2024). The GWAS for IDPs was based on 39,691 UK Biobank participants (Smith et al., 2021). For fiber radial diffusivity IDPs, instrumental variables were selected from the GWAS of the corresponding fiber’s λ2 IDPs. Independent single-nucleotide polymorphisms (SNPs) were identified as instruments using the following criteria: at genome-wide significance (p < 5 × 10^−8^) with neuroticism, minor allele frequency ≥0.01, and linkage disequilibrium R^2^ < 0.001 within a 10,000 kb clumping window. SNPs were harmonized to ensure their effects on neuroticism and IDPs corresponded to the same allele, with palindromic variants with a minor allele frequency above 0.42 or unknown excluded.

### Statistical analyses

We used linear regression to examine the association between the neuroticism z-score and each of the 1,747 IDPs, adjusting for all preselected covariates. To account for multiple testing, Bonferroni corrections were applied, and IDPs meeting the adjusted significance threshold (*p* < 2.86 × 10^−5^) were considered for further analyses.

We performed causal mediation analyses (Kosuke, Luke, & Teppei, 2010) to evaluate whether the observed associations were mediated by preselected health conditions. This approach decomposed the total effect of neuroticism on IDPs into two components: (1) the average causal mediation effect (ACME), which quantifies how much the IDP changes due to the health condition, while keeping neuroticism constant; and (2) the average direct effect, which measures how much the IDP changes directly due to neuroticism, holding the health condition status constant. The proportion mediated was calculated as ACME/total effect. These estimates were derived from a logistic regression model, where each health condition was regressed on neuroticism and preselected covariates, and a linear regression model, where each IDP was regressed on neuroticism, the health condition, and the same covariates. To account for multiple testing, a Bonferroni adjustment was applied to the significance level of the ACME. Detailed methods for the mediation analysis are provided in the Supplementary methods.

We conducted two-sample Mendelian randomization (MR) to investigate bidirectional causal relationships between neuroticism and IDPs that showed significant observational associations with neuroticism. The primary MR analysis used the inverse-variance weighted (IVW) method. For significant associations (uncorrected *p* < 0.05), we conducted sensitivity analyses to assess the robustness of causal inference and address potential bias from sample overlap or horizontal pleiotropy (Supplementary Methods).

All statistical analyses were conducted in the R software (version 4.2.2) using the TwoSampleMR (Hemani et al., 2018; Hemani, Tilling, & Davey Smith, 2017), MR-PRESSO (Verbanck, Chen, Neale, & Do, 2018), MRlap (Mounier & Kutalik, 2023), and mediation (Tingley, Yamamoto, Hirose, Keele, & Imai, 2014) packages.

## Results

### Sample characteristics

The mean age of the 36,901 participants was 64.4 years (SD = 7.7), and 51.4% were female (Table 1). Neuroticism was measured at the imaging visit using the 12-item Eysenck Personality Questionnaire-Revised Short Form (EPQ-RS; score range 0–12). Scores were skewed towards lower values, with one-third of participants scoring between 0 and 1, and two-thirds scoring below 4 (Supplementary Figure S1). Those with higher levels of neuroticism tended to be younger, female, have lower socioeconomic status, consume less alcohol, and be current smokers. Additionally, histories of depression, anxiety, and hypertension were more common among participants with higher neuroticism, while histories of IHD and diabetes were similar across different levels of neuroticism.

### Observational associations with brain structure

**Figure 1:**
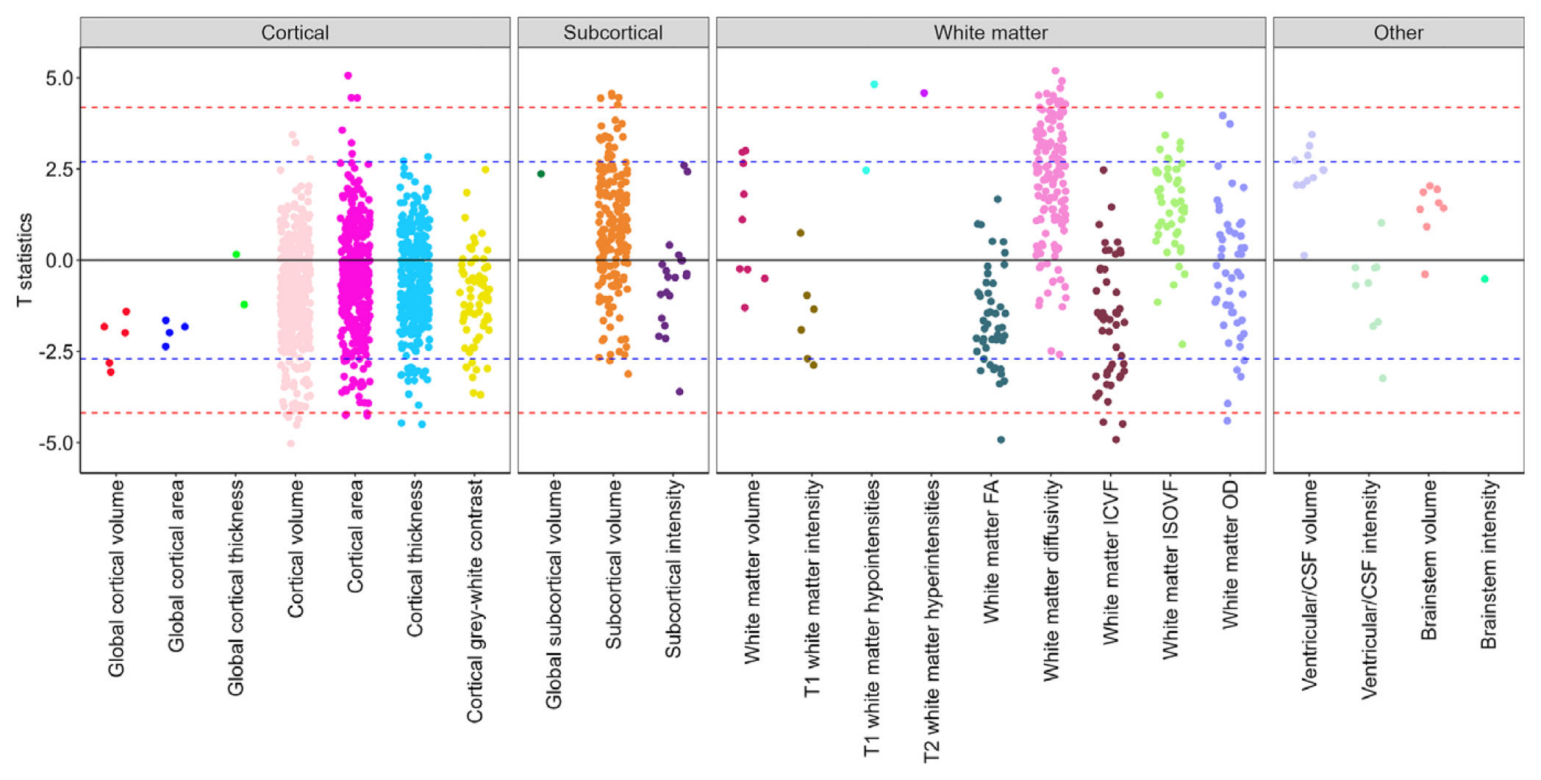
Brain-wide association between neuroticism and imaging-derived phenotypes (IDPs) by structural group. T statistics for the linear regression between neuroticism and brain-wide IDPs. Red dashed line indicates the Bonferroni threshold (1,747 tests, p = 2.86 × 10^−5^, T statistics = ±4.18) and blue dashed line indicated the False Discovery rate threshold (1,747 tests, p = 0.007, T statistics = ±2.70). See Supplementary Table S5 for regression coefficients and 95% confidence intervals. FA, ‘fractional anisotropy’; ICVF, ‘intra-cellular volume fraction’; ISOVF, ‘isotropic volume fraction’; OD, ‘orientation dispersion’; CSF, ‘cerebrospinal fluid’.

Associations between neuroticism scores and brain structure were examined in a brain-wide study across multiple neuroimaging modalities. Among 1,747 imaging-derived phenotypes (IDPs), 40 showed significant associations with neuroticism after Bonferroni correction (*p* < 2.86 × 10^−5^) (Figure 1; Supplementary Table S5), with the strongest associations observed for regional cortical and subcortical volumes and white matter macro- and microstructure.

For cortical volume, higher neuroticism was most strongly associated with reduced volume in the frontal lobe (left frontal medial cortex, left subcallosal cortex, and left medial orbitofrontal cortex) and the bilateral anterior cingulate cortex in the limbic lobe (Table 2). Neuroticism was also associated with increased surface area in the left parahippocampal gyrus (temporal lobe), and reduced area in the left pericalcarine cortex and calcarine sulcus (occipital lobe). Conversely, neuroticism showed a negative association with cortical thickness in the left parahippocampal gyrus. These associations were consistent across IDPs derived from different segmentation pipelines and were observed bilaterally, though generally stronger in the left hemisphere (Supplementary Figures S2–S5).

In subcortical regions, neuroticism was associated with larger volumes of the bilateral caudate, putamen, and ventral striatum (Table 2; Supplementary Figure S6).

**Figure 2:**
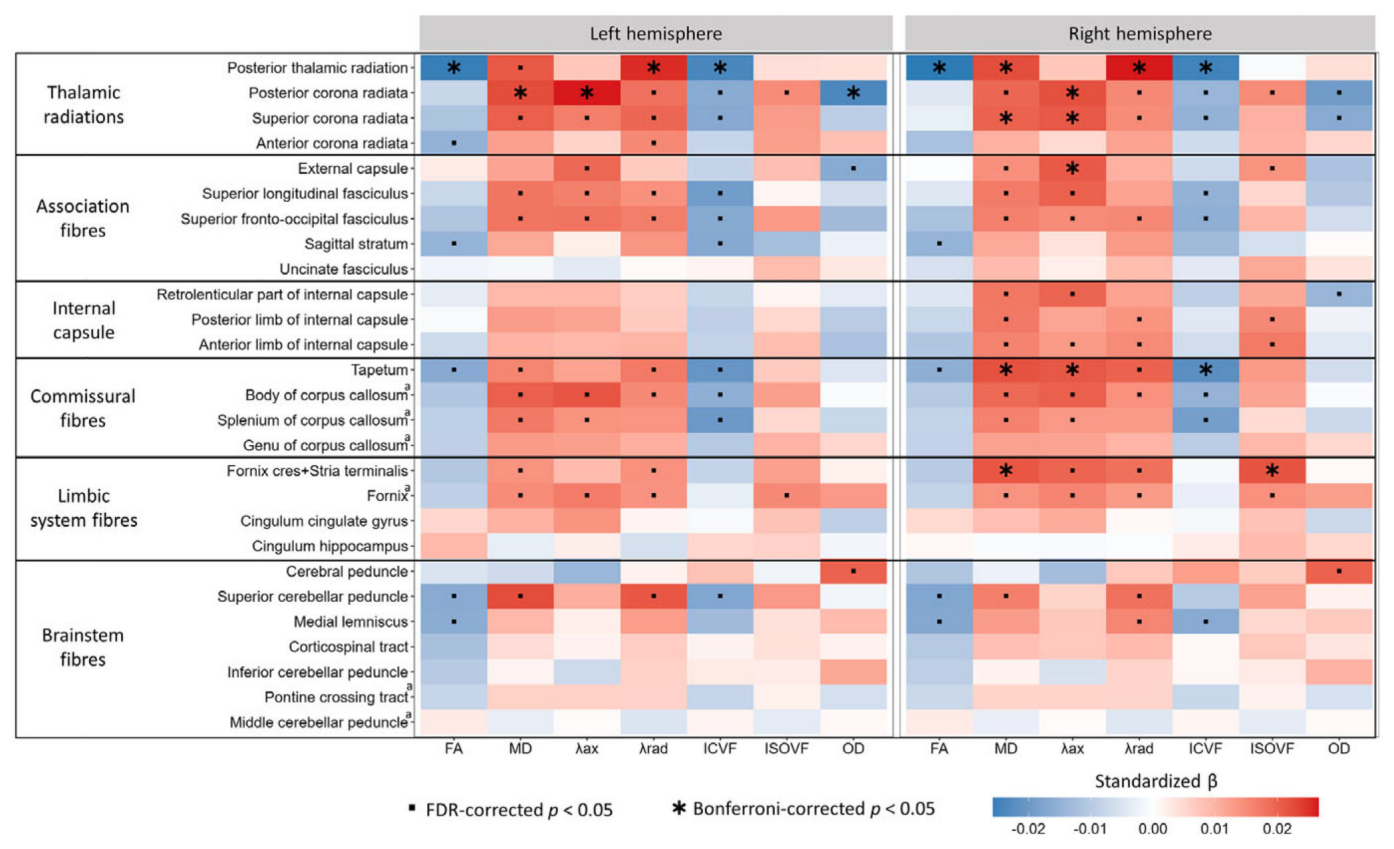
Associations between neuroticism and white matter microstructure indices across white matter tract regions. ^a^Structures with only a global measure (left and right hemisphere estimates are duplicated). Asterisks indicate Bonferroni-adjusted significant associations (*p* < 2.86 × 10^−5^); squares indicate FDR-adjusted significance at the same threshold (*p* < 0.007). FA, ‘Fractional Anisotropy’; λax, ‘Axial Diffusivity’; λrad, ‘Radial Diffusivity’; MD, ‘Mean Diffusivity’; ICVF, ‘Intra-Cellular Volume Fraction’; ISOVF, ‘Isotropic Volume Fraction’; OD, ‘Orientation Dispersion’.

Neuroticism was also associated with markers of poorer white matter macrostructure, including higher T1 hypointensities and T2 hyperintensities, both of which reached Bonferroni-corrected significance (Figure 1). White matter microstructural metrics showed the strongest associations with neuroticism across all imaging modalities. Neuroticism was consistently associated with lower fractional anisotropy (FA), higher diffusivity (mean diffusivity [MD], axial diffusivity [λax], and radial diffusivity [λrad]), lower intracellular volume fraction (ICVF) and, to a lesser extent, higher isotropic volume fraction (ISOVF) across multiple tracts (Figures 1 and 2), all reflecting a pattern of poorer white matter microstructure. No consistent trend was observed for orientation dispersion (OD) across tracts.

The most robust findings were localized to the thalamic radiations, with poorer microstructure in the bilateral posterior thalamic radiation, posterior corona radiata, and superior corona radiata reaching Bonferroni-corrected significance (Figure 2). Additional Bonferroni-corrected significant associations included increased diffusivity and decreased ICVF in the right tapetum (commissural fibers), increased diffusivity and ISOVF in the fornix cres/stria terminalis (limbic system fibers), and increased diffusivity in the external capsule (association fibers). Patterns were generally similar across hemispheres but tended to be stronger on the right (Figure 2; Supplementary Figure S7).

No Bonferroni-significant associations were observed for ventricular volume, brainstem volume, or brainstem intensity (Figure 1).

### Mediation by health conditions

**Figure 3:**
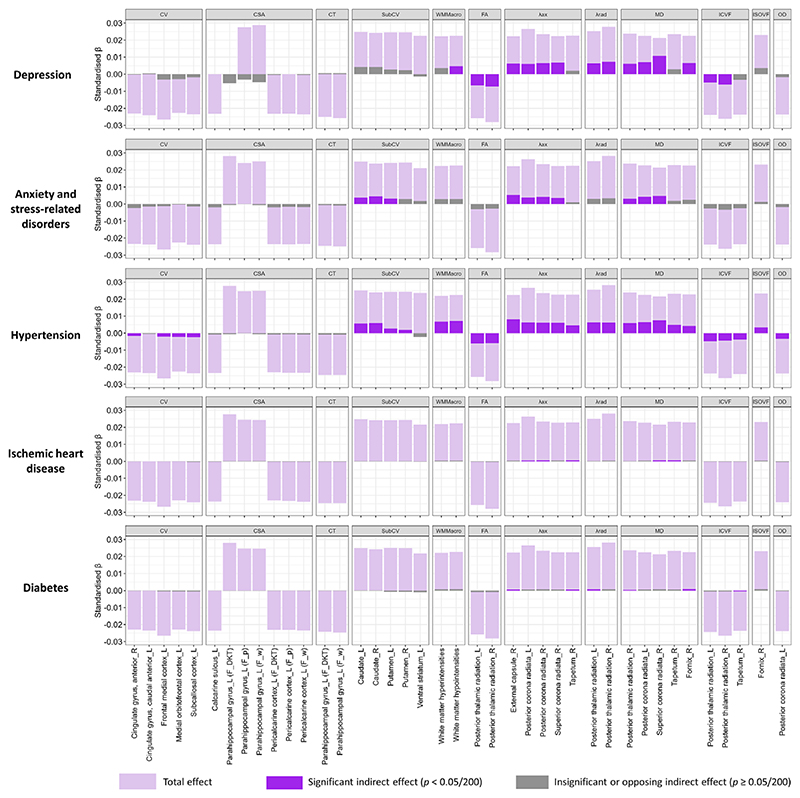
Total effect of neuroticism on significant imaging-derived phenotypes (IDPs) and indirect effects mediated by preselected health conditions. Light purple bars represent the total effect, dark purple bars indicate significant (Bonferroni-adjusted) indirect (mediating) effects, and grey bars denote insignificant (Bonferroni-adjusted) or directionally inconsistent indirect (mediating) effects. See Supplementary Table S2 for regression coefficients and mediation proportions. CV, ‘Cortical Volume’; CSA, ‘Cortical Surface Area’; CT, ‘Cortical Thickness’; subCV, ‘Subcortical Volume’; G/W, ‘Grey-White Matter Contrast’; FA, ‘Fractional Anisotropy’; λax, ‘Axial Diffusivity’; λrad, ‘Radial Diffusivity’; MD, ‘Mean Diffusivity’; ICVF, ‘Intra-Cellular Volume Fraction’; ISOVF, ‘Isotropic Volume Fraction’; OD, ‘Orientation Dispersion’.

We explored potential mediating pathways underlying the observed associations between neuroticism and brain structure using causal mediation analysis. This method decomposes effects into direct and mediation effects. We hypothesized that vascular and mental conditions could be important factors, given their links with both dementia and neuroticism. Furthermore, given the availability of therapeutic interventions, identification as mediators would have important implications for the mitigation of neuroticism-related brain changes. Overall, the examined conditions explained a substantial proportion of the associations with white matter macro- and microstructure, smaller proportions for cortical and subcortical volumes, and no mediation for cortical surface area or thickness (Figure 3).

Among the examined conditions, hypertension showed the most consistent mediating effects across structures. The largest average causal mediation effect (ACME) was observed for the λax in the right external capsule (ACME = 0.008, 95% CI [0.007, 0.010]; proportion mediated = 36%; Supplementary Table S6). Other regions with mediation proportions >30% included the MD in the right superior corona radiata (35%), T1 hypointensities (31%), and T2 hyperintensities (31%). Mediation proportions for associations with other white matter structures were ≥ 15%, while for the right and left caudate volume, they were 25% and 22%, respectively. For other cortical/subcortical volumes, mediation proportions were mostly <10%.

Depression did not mediate cortical/subcortical volume but strongly mediated white matter microstructure, with proportions generally >20%. Notably, it mediated 51% of the association with MD in the right superior corona radiata (ACME = 0.011, 95% CI [0.008, 0.013]), 30% for λax in the same region, and 32% for MD in the left posterior corona radiata. Anxiety mediated fewer white matter tracts and with smaller proportions (9%–24%); it also mediated associations with caudate volume (15% for the left and 19% for the right). IHD and diabetes mediated only a few associations with white matter microstructure, with smaller proportions (2%–4%).

### Genetic associations with brain structure

**Figure 4:**
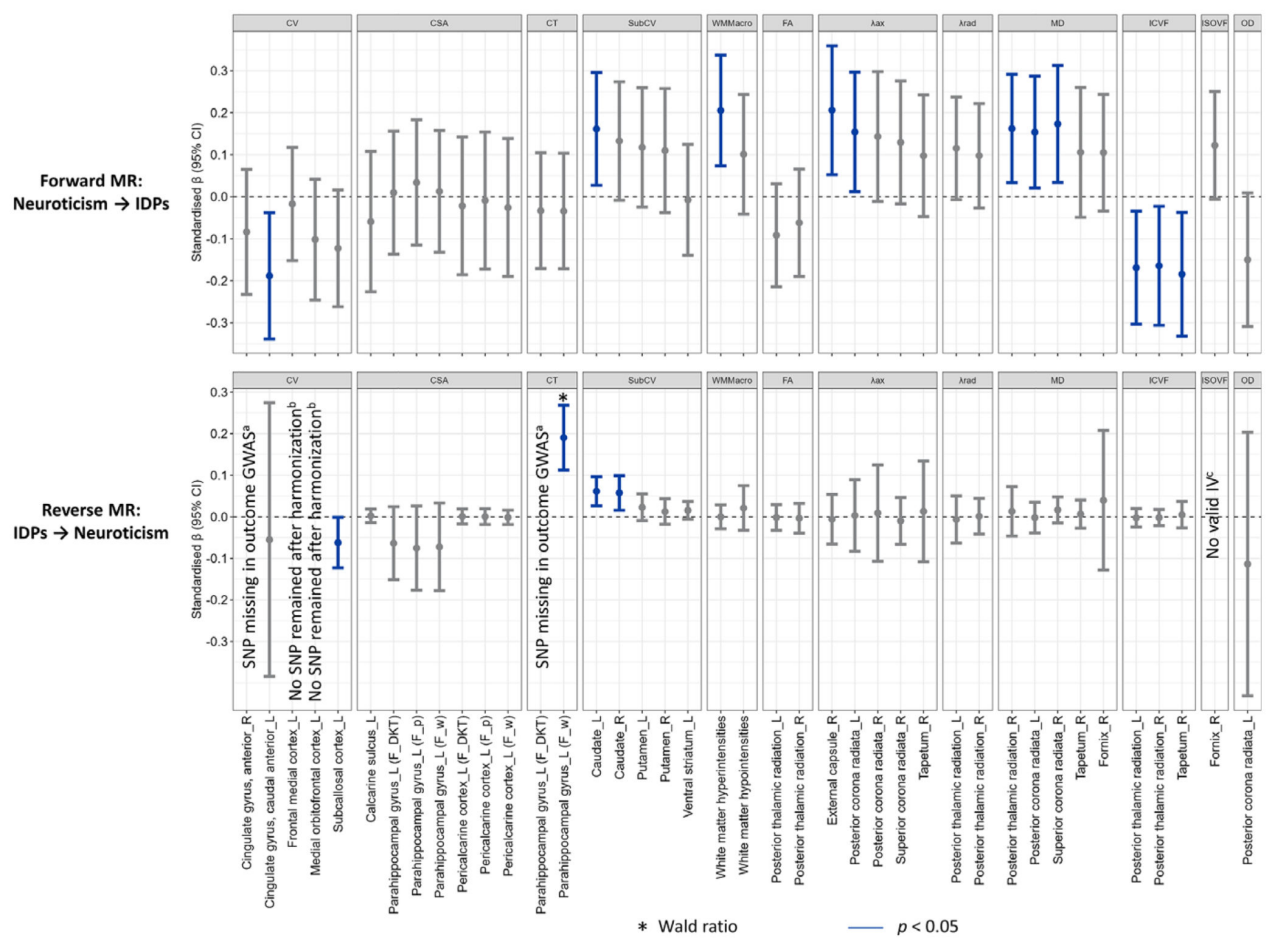
Inverse-variance weighted (IVW) estimates for the bidirectional Mendelian randomization. ^a^Only one genome-wide significant and independent SNP identified, but it was not available in the outcome GWAS; ^b^No SNP remained after harmonization due to all being palindromic with minor allele frequency > 0.42 or unknown; ^c^No genome-wide significant and independent SNPs identified for use as IVs. IV, instrumental variable; SNP, single nucleotide polymorphism; GWAS, ‘genome-wide association studies’; ‘F_DKT’, ‘derived from FreeSurfer DKT atalas’; ‘F_w’, ‘derived from FreeSurfer Desikan white atlas’; ‘F_w’, ‘derived from FreeSurfer Desikan pial atlas’. See Supplementary Tables S8 and Table S10 for regression coefficients and 95% confidence intervals.

To disentangle the direction of observed associations between neuroticism and brain structure, we used bidirectional Mendelian randomization (MR). One hundred and nineteen independent genetic variants identified in a large GWAS were used to instrument neuroticism (Supplementary Table S7). From the 40 IDPs that showed Bonferroni-corrected significant associations with neuroticism in the observational analysis, 11 had significant genetic associations in forward MR (Figure 4).

Genetically predicted higher neuroticism was associated with a larger volume of the left caudate (inverse-variance weighted [IVW] *β* = 0.16, p = 0.018; Supplementary Table S8), as well as with microstructural differences in several white matter tracts, including higher λax in the right external capsule (*β* = 0.21, *p* = 0.008), and both higher λax (*β* = 0.15, *p* = 0.034) and MD (*β* = 0.15, *p* = 0.024) in the left posterior corona radiata. These associations were robust across sensitivity analyses accounting for pleiotropy and sample overlap (Supplementary Table S9). Additionally, significant IVW associations were observed with lower volume of the left anterior cingulate gyrus and higher white matter hyperintensities. While sensitivity analyses for these associations aligned with the direction of effects estimated by IVW, potential pleiotropy was detected (all p-values for Egger intercept <0.05 and p-values for MR Egger >0.05). For white matter hyperintensities, no outlier SNPs were detected by MR-PRESSO or through diagnostic plots (Supplementary Figure S8), whereas one outlier SNP was identified for the left anterior cingulate gyrus but its removal did not materially alter the results. Steiger filtering indicated stronger instrument-exposure than instrument-outcome correlations in all cases, except for the left caudate and anterior cingulate gyrus volumes, where the differences were not statistically significant (*p*_Steiger_ > 0.05), supporting the assumption of correct causal direction.

In reverse MR, genetically predicted larger volumes of the left and right caudate and lower volume of the left subcallosal cortex (instrumented using 8, 7, and 5 SNPs, respectively; Supplementary Table S10), were associated with higher neuroticism. However, none of these associations remained significant in sensitivity analyses accounting for sample overlap, and all showed evidence of pleiotropy (Supplementary Table S9). A genetically predicted larger thickness of the left parahippocampal gyrus was associated with higher neuroticism (Wald ratio = 0.19, *p* < 10^−5^), but this finding was based on a single instrumental SNP.

## Discussion

In this large study of mid- to older-aged adults, neuroticism was associated with a broad range of structural brain differences. The most significant associations were observed in regions implicated in cognitive impairment and dementia, including reduced volume and thickness in key cortical areas, along with indicators of cerebrovascular burden such as increased white matter hyperinten-sities and diffusivity in white matter tracts involved in memory and cognition. White matter macro- and microstructural differences were largely mediated by depression, anxiety, and hypertension, whereas cortical and subcortical volume associations were only modestly mediated by hypertension. MR analyses suggested that these structural differences may be a consequence of, rather than a vulnerability to, high neuroticism.

Previous studies of neuroticism and cortical structure in mid- to older-aged adults, based on validated personality questionnaires, have been underpowered due to small sample sizes (*N* = 29–578) and reliance on voxel- or vertex-wise rather than IDP analyses. (Bjørnebekk et al., 2013; Kapogiannis et al., 2013; Lewis et al., 2018; Li et al., 2017; Taki et al., 2013; Tuerk et al., 2016; Wright, Feczko, Dickerson, & Williams, 2007) While some reported no significant associations (Lewis et al., 2018; Li et al., 2017; Taki et al., 2013), others identified several cortical regions (Bjørnebekk et al., 2013; Kapogiannis et al., 2013; Tuerk et al., 2016; Wright et al., 2007). Our findings align with the most consistent result in the literature: reduced volume in the ventromedial prefrontal cortex (vmPFC) (Kapogiannis et al., 2013; Tuerk et al., 2016). This region includes the structures showing the strongest negative associations in our study—the frontal medial cortex, subcallosal cortex, anterior cingulate cortex, and medial orbitofrontal cortex—as well as those reported in prior work, such as the superior and inferior orbitofrontal gyri (Kapogiannis et al., 2013). Additionally, another study reported negative associations with the surface area of many of these vmPFC regions (Bjørnebekk et al., 2013. vmPFC is critical for higher cognition (learning, social cognition, and decision-making) (Apps, Rushworth, & Chang, 2016; Hiser & Koenigs, 2018) and is a neuroimaging hallmark of frontotemporal dementia (Wong, Flanagan, Savage, Hodges, & Hornberger, 2014).

The vmPFC is closely connected with the hippocampus and parahippocampal gyrus (Nieuwenhuis & Takashima, 2011), structures whose reduced volume and thinning serve as early biomarkers for AD (Echávarri et al., 2011; Krumm et al., 2016). While a previous study reported an association between neuroticism and reduced parahippocampal volume (Kapogiannis et al., 2013), we instead found a pattern of increased surface area but reduced thickness—consistent with evidence that age-related volume changes in this region are primarily driven by thinning rather than surface area loss (Storsve et al., 2014). The parahippocampal gyrus is located in the temporal lobe, where prior studies have reported reduced volume in association with neuroticism (Kapogiannis et al., 2013; Tuerk et al., 2016). These temporal lobe findings were not replicated in our analysis, potentially due to differences in cortical atlases, covariate adjustment, or participant characteristics. Instead, we identified a novel negative association between neuroticism and surface area in the calcarine and pericalcarine cortices—regions previously reported as affected in patients with AD (Ahmad, Javed, Athar, & Shahzadi, 2023; Hartikainen et al., 2012).

For subcortical structures, we observed positive associations between neuroticism and volumes of the caudate and putamen (which together form the dorsal striatum) as well as the ventral striatum. These structures were not highlighted in previous whole-brain studies (Chen & Canli, 2022), likely due to insufficient power. The relationship between striatal volume and brain health remains controversial. While larger striatal volumes have been linked to better cognitive performance in cross-sectional studies (Bauer, Toepper, Gebhardt, Gallhofer, & Sammer, 2015; Grazioplene et al., 2015), longitudinal data suggest they predict higher dementia risk (van der Velpen et al., 2023), potentially reflecting early compensatory changes before broader atrophy emerges (de Jong et al., 2008).

The strongest and most widespread associations were observed in white matter, which is expected given neuroticism’s established links to vascular conditions such as cardiovascular disease (Zhang et al., 2021) and vascular dementia (Gao et al., 2024; Terracciano et al., 2021). Neuroticism was associated with increased white matter hyperintensities—markers of small vessel disease or vascular pathology (Debette & Markus, 2010)—consistent with prior findings (Terracciano et al., 2023). Previous hypothesis-free studies of neuroticism and diffusion MRI have been limited by small sample sizes and a focus on FA (and occasionally diffusivity metrics), but their results support our findings: the most significant microstructural differences in our analysis were located in the thalamic radiations, consistent with a previous study reporting differences in fiber tracts connecting thalamic nuclei to the frontal lobes (Bjørnebekk et al., 2013, while adding greater anatomical specificity. Other associations, including with the external capsule and tapetum, also replicate prior work (McIntosh et al., 2013; Xu & Potenza, 2012), whereas our finding in the fornix cres/stria terminalis appears novel. These tracts are known to be vulnerable to vascular brain aging (Habes et al., 2018) and degenerate early in Alzheimer’s progression (Bendlin et al., 2010; Mayo, Mazerolle, Ritchie, Fisk, & Gawryluk, 2017; Mielke et al., 2012; Ringman et al., 2007; Schumacher et al., 2022; Sun et al., 2014; Yin et al., 2015; Yu, Lam, & Lee, 2017). To our knowledge, this is the first study to extend beyond conventional DTI measures to examine NODDI metrics, which suggested that these diffusivity changes primarily reflect axonal/dendritic loss (indicated by ICVF) rather than excess extracellular water (ISOVF) or tract disorganization (OD) (Zhang et al., 2012).

Hypertension mediated both cortical and white matter abnormalities, likely through mechanisms such as vascular injury, reduced cerebral perfusion, and neuroinflammation, all of which contribute to neuronal death involving cell bodies, axons, and dendrites (Baggeroer, Cambronero, Savan, Jefferson, & Santisteban, 2024; Heneka et al., 2015; Rao, Kellom, Kim, Rapoport, & Reese, 2012). In contrast, depression and anxiety primarily mediated white matter microstructural changes, but not cortical alterations. This aligns with prior studies showing that polygenic risk for depression is associated with white matter abnormalities but not cortical structure (Flinkenflügel et al., 2024; Shen et al., 2020). A proposed mechanism involves hyperactivity of the hypothalamus–pituitary–adrenal axis in depression (Pariante, 2009), where glucocorticoid toxicity may preferentially affect axons/dendrites (Jauregui-Huerta et al., 2010). IHD showed minimal mediation (likely due to low prevalence [5.8%] in our sample), while diabetes—included as ‘negative control’ given its lack of association with neuroticism—demonstrated no mediating effects as expected.

MR analyses provided further support for a causal link from neuroticism to white matter microstructural alterations, particularly increased diffusivity and reduced ICVF in the thalamic radiations. We also observed bidirectional associations between neuroticism and caudate volume, though reverse MR analyses suggested potential horizontal pleiotropy, where genetic variants may influence neuroticism through pathways independent of caudate volume. For other genetically predicted IDPs, associations with neuroticism were generally null. However, it may be premature to establish a definitive unidirectional relationship from neuroticism to brain structures. This is primarily due to the small number of genome-wide significant instruments currently available for neuroimaging traits, which reduces power to detect causal associations (Shen et al., 2020).

To our knowledge, this study is the largest and most comprehensive investigation of neuroticism’s associations with brain structure, using multimodal neuroimaging metrics and a hypothesis-free approach. By applying causal mediation analyses and MR, we provide the first evidence of potential mediators and directionality, offering new insights into neuroticism’s biological underpinnings and clinical management.

However, this study has several limitations. First, UK Biobank participants, particularly those who participated in the imaging assessment (Cox et al., 2019), tend to be healthier and of higher socioeconomic status than the general population (Fry et al., 2017), which may introduce selection bias and limit the generalizability of our findings. Second, while neuroticism is generally stable across adulthood, the cross-sectional nature of neuroticism and MRI measurements precludes firm conclusions about temporal sequence, and residual confounding in observational analyses remains likely. Though we employed MR to strengthen causal inference, it remains subject to bias from weak instruments and horizontal pleiotropy. Third, mediation analyses also relied on cross-sectional data, where mediators and outcomes were measured simultaneously; furthermore, potential mediators (health conditions) were captured through self-reported diagnoses or hospital inpatient records, potentially missing mild cases or symptoms and introducing misclassification bias.

In conclusion, neuroticism is associated with a broad profile of structural brain differences indicative of poorer brain health. These include reduced frontal/limbic volumes, cortical thinning in memory-related regions, striatal enlargement, and widespread white matter abnormalities. Hypertension, depression, and anxiety appear to mediate many of these associations. These findings highlight shared biological pathways linking neuroticism, cognitive function, and dementia risk. While neuroticism itself remains stable in adulthood, targeting our identified mediators—that is, addressing vascular and mental health, may lessen the potential impact of neuroticism on the brain.

## Supplementary Material

Supplementary Information

## Figures and Tables

**Table 1:** Characteristics of participants

Characteristics^a^	Overall	Neuroticism score tertile		
		1 (score 0–1)	2 (score 2–4)	3 (score 5–12)
*n*	36,901	13,341	11,829	11,731
Age (mean (SD))	64.44 (7.71)	65.47 (7.58)	64.67 (7.64)	63.03 (7.72)
Sex (%)				
Female	18,971 (51.4)	5,546 (41.6)	6,366 (53.8)	7,059 (60.2)
Male	17,930 (48.6)	7,795 (58.4)	5,463 (46.2)	4,672 (39.8)
Townsend deprivation index quintile (%)				
1 (Least deprived)	8,915 (24.2)	3,319 (24.9)	2,933 (24.8)	2,663 (22.7)
2	8,491 (23.0)	3,096 (23.2)	2,779 (23.5)	2,616 (22.3)
3	7,680 (20.8)	2,805 (21.0)	2,422 (20.5)	2,453 (20.9)
4	6,879 (18.6)	2,409 (18.1)	2,163 (18.3)	2,307 (19.7)
5 (Most deprived)	4,936 (13.4)	1,712 (12.8)	1,532 (13.0)	1,692 (14.4)
Education (%)				
Primary	2,195 (5.9)	769 (5.8)	673 (5.7)	753 (6.4)
Secondary	7,001 (19.0)	2,239 (16.8)	2,194 (18.5)	2,568 (21.9)
Post-secondary non-tertiary	4,121 (11.2)	1,481 (11.1)	1,403 (11.9)	1,237 (10.5)
Tertiary	23,584 (63.9)	8,852 (66.4)	7,559 (63.9)	7,173 (61.1)
Ethnicity (%)				
Non-white	1,025 (2.8)	427 (3.2)	306 (2.6)	292 (2.5)
White	35,876 (97.2)	12,914 (96.8)	11,523 (97.4)	11,439 (97.5)
Body mass index category (%)				
Normal (<25 kg/m^2^)	14,839 (40.2)	5,172 (38.8)	4,940 (41.8)	4,727 (40.3)
Overweight (25–29.9 kg/m^2^)	15,255 (41.3)	5,789 (43.4)	4,810 (40.7)	4,656 (39.7)
Obese (≥30 kg/m^2^)	6,807 (18.4)	2,380 (17.8)	2,079 (17.6)	2,348 (20.0)
Alcohol intake (%)				
Less than 4 times per week	30,588 (82.9)	10,970 (82.2)	9,830 (83.1)	9,788 (83.4)
Drinking alcohol daily or almost daily	6,313 (17.1)	2,371 (17.8)	1,999 (16.9)	1,943 (16.6)
Smoking (%)				
Never	23,221 (62.9)	8,586 (64.4)	7,494 (63.4)	7,141 (60.9)
Previous	12,436 (33.7)	4,343 (32.6)	3,944 (33.3)	4,149 (35.4)
Current	1,244 (3.4)	412 (3.1)	391 (3.3)	441 (3.8)
Disease history (%)^b^				
Depression	3,611 (9.8)	441 (3.3)	857 (7.2)	2,313 (19.7)
Anxiety and stress disorders	1,761 (4.8)	206 (1.5)	403 (3.4)	1,152 (9.8)
Ischemic heart disease	2,150 (5.8)	790 (5.9)	701 (5.9)	659 (5.6)
Hypertension	10,719 (29.0)	3,746 (28.1)	3,454 (29.2)	3,519 (30.0)
Diabetes	1,882 (5.1)	715 (5.4)	549 (4.6)	618 (5.3)

a Townsend deprivation index and ethnicity were measured at baseline, other characteristics were measured at the imaging visit.

b These conditions were ascertained through verbal interview and linked hospital inpatient records preceding the imaging visit, with diagnostic codes detailed in Supplementary Table S4.

**Table 2:** Summary of associations between neuroticism and cortical/subcortical structures after Bonferroni correction (*p* < 2.9 × 10^−5^)

Region	Structure	Hemisphere	Structural measures	Brain segmentation tools	Standardised *β*	SE	*p*
**Cortical structure**							
Temporal	Parahippocampal gyrus	Left	Area	Freesurfer DKT	0.028	0.005	4.2 × 10^–7^
Frontal	Frontal medial cortex	Left	Volume	FAST	–0.027	0.005	5.1 × 10^–7^
Frontal	Subcallosal cortex	Left	Volume	FAST	–0.024	0.005	6.3 × 10^–6^
Temporal	Parahippocampal gyrus	Left	Thickness	Freesurfer desikan white	–0.025	0.005	6.8 × 10^–6^
Temporal	Parahippocampal gyrus	Left	Thickness	Freesurfer DKT	–0.025	0.005	8.0 × 10^–6^
Temporal	Parahippocampal gyrus	Left	Area	Freesurfer desikan pial	0.024	0.005	8.6 × 10^–6^
Temporal	Parahippocampal gyrus	Left	Area	Freesurfer desikan white	0.024	0.005	8.8 × 10^–6^
Limbic	Cingulate gyrus, caudal anterior	Left	Volume	Freesurfer DKT	–0.024	0.005	1.3 × 10^–5^
Limbic	Cingulate cortex, anterior	Right	Volume	Freesurfer a2009s	–0.023	0.005	1.7 × 10^–5^
Occipital	Pericalcarine cortex	Left	Area	Freesurfer desikan white	–0.024	0.006	2.1 × 10^–5^
Occipital	Calcarine sulcus	Left	Area	Freesurfer a2009s	–0.023	0.006	2.2 × 10^–5^
Frontal	Medial orbitofrontal cortex	Left	Volume	Freesurfer DKT	–0.023	0.005	2.3 × 10^–5^
Occipital	Pericalcarine cortex	Left	Area	Freesurfer desikan pial	–0.023	0.006	2.3 × 10^–5^
Occipital	Pericalcarine cortex	Left	Area	Freesurfer DKT	–0.023	0.006	2.8 × 10^–5^
**Subcortical structure**							
Striatum	Caudate	Left	Volume	FAST	0.025	0.005	5.0 × 10^–6^
Striatum	Putamen	Right	Volume	FAST	0.024	0.005	7.0 × 10^–6^
Striatum	Putamen	Left	Volume	FAST	0.024	0.005	8.4 × 10^–6^
Striatum	Caudate	Right	Volume	FAST	0.024	0.005	9.1 × 10^–6^
Striatum	Ventral striatum	Left	Volume	FAST	0.021	0.005	2.1 × 10^–5^

Significant associations between neuroticism and cortical/subcortical structures after Bonferroni correction (1,747 tests; *p* < 2.9 × 10^−5^). Results were ranked by uncorrected p-values. See Supplementary Table S5 for regression coefficients and 95% confidence intervals. FAST, ‘FMRIB’s Automated Segmentation Tool’.
