# Supplementary material for: Association between neuroticism and brain-wide structural outcomes: Mediation by vascular and mental conditions
Source: Psychol Med. 2025 Nov 14;55:e343. doi: 10.1017/S0033291725102390 (PMC7618729; doi:10.1017/S0033291725102390)
Supplement: Gao et al. supplementary material 1 — Gao et al. supplementary material [file S0033291725102390sup001.docx]

Association between neuroticism and brain-wide structural outcomes: mediation by vascular and mental conditions

Supplementary Materials

Contents

[Supplementary methods 2](#_Toc193638541)

[Figure S1. Histogram of neuroticism scores 4](#_Toc193638542)

[Figure S2. Association between neuroticism and regional cortical volumes 5](#_Toc193638543)

[Figure S3. Association between neuroticism and regional cortical area 6](#_Toc193638544)

[Figure S4. Association between neuroticism and regional cortical thickness 7](#_Toc193638545)

[Figure S5. Association between neuroticism and regional grey-white matter contrast 8](#_Toc193638546)

[Figure S6. Association between neuroticism and subcortical volumes 9](#_Toc193638547)

[Figure S7. Association between neuroticism and white matter microstructure across tracts 10](#_Toc193638548)

[Figure S8. Scatter plots and funnel plots of Mendelian randomization analyses for the association between neuroticism and IDPs with significant inverse variance weighted estimates 11](#_Toc193638549)

[Figure S9. Scatter plots and funnel plots of Mendelian randomization analyses for the association between IDPs and neuroticism with significant inverse variance weighted estimates 15](#_Toc193638550)

[Reference 16](#_Toc193638551)

# Supplementary methods

*Structural imaging-derived phenotypes (IDPs)*

T1-weighted imaging allows precise volumetric measurements of cortical and subcortical regions. Using T1-weighted data, FMRIB’s Automated Segmentation Tool (FAST)^1^ segmented brain tissue into cerebrospinal fluid, grey matter, and white matter. 139 cortical regions of interest (ROIs) were defined using the MNI152 template, combining parcellations from Harvard-Oxford cortical and subcortical atlases and the Diedrichsen cerebellar atlas. Grey matter volumes in these ROIs were summed to create 139 IDPs representing cortical grey matter volume. 15 subcortical structures' shapes and volumes were modelled using FMRIB’s Integrated Registration and Segmentation Tool (FIRST).^2^ The T1 images are also processed with FreeSurfer based on different brain atlases (e.g., a2009s, BA exvivo, desikan white, and DKT).

Diffusion MRI (dMRI) reflects white matter microstructure by measuring how water molecules move within tissue. Water molecules show greater directional coherence and reduced diffusion when fibres are densely packed, with undamaged myelin and cell membranes. dMRI generates three diffusivity parameters (λ1, λ2, and λ3). Axial diffusivity (λax = λ1) reflects diffusion along the axonal fibres. In contrast, radial diffusivity (λrad = (λ2 + λ3)/2) represents the average diffusion perpendicular to the fibres. Fractional Anisotropy (FA) measures the directional coherence of water diffusion along axonal fibres, while Mean Diffusivity (MD) provides an average of all diffusion directions. dMRI data was also fed into Neurite orientation dispersion and density imaging (NODDI)^3^ modelling, which provides estimates of neurite density (intra-cellular volume fraction; ICVF), extra-cellular water diffusion (isotropic volume fraction; ISOVF), and tract complexity/fanning (orientation dispersion index, OD).

*Mendelian randomization*

MR-Egger,^4^ weighted median methods,^5^ weighted mode-based estimation,^6^ and robust adjusted profile score methods^7^ were used to evaluate the consistency of the causal inference. We applied the MR-PRESSO test to identify potential outliers and obtain corrected estimates by removing them.^8^ Given both the GWAS for neuroticism and IDPs were derived, at least in part, from UK Biobank participants, we conducted a sensitivity analysis using "MRlap" to adjust for potential bias from partially overlapping samples.^9^ For each pair of traits, we confirmed that genetic instruments had stronger associations with the exposure than with the outcome using Steiger filtering,^10^ assessed heterogeneity using Cochran’s Q-statistic, and tested for pleiotropy using the Egger intercept. We also visually assessed heterogeneity using scatter plots and funnel plots.^11^

Power calculations were performed using an online calculator.^12^ With an R² value of 0.009, a significance level of 0.05, and a sample size of 39,691 individuals for the outcome GWAS,^13^ the analysis had 80% power to detect a causal effect of a 1-SD change in neuroticism resulting in a 0.15-SD change in the IDP.

*Mediation*

We used causal mediation analysis^14^ to estimate the proportion of the association between neuroticism and the IDP that is mediated by health conditions. This approach decomposes the total effect (TE) of neuroticism on the IDP (Y) into direct effects (DE) and causal mediation effects (CME) through disease conditions (M). We fit the following generalized linear models: (1) a mediator model, where each health condition (M) was modeled as a binary outcome using logistic regression, regressed on neuroticism (X) and preselected covariates; and (2) an outcome model, where IDP (Y) was modeled as a continuous outcome using linear regression, regressed on neuroticism (X), the mediator (M), and the same set of covariates. Based on the regression coefficients from these models, we estimated the average direct effect (ADE = E[Y(a,M(a*)) – Y(a*,M(a*))]) and average causal mediation effect (ACME = E[Y(a,M(a)) – Y(a,M(a*))]), where a ≠ a*, and Y(*a,m*) represents the expected outcome if a participant had a neuroticism level *a* and a disease history status *m* (existence or absence of the disease). 95% confidence intervals for the ACME were derived using a quasi-Bayesian Monte Carlo approach with 1000 simulations. For ACME that were statistically significant after Bonferroni correction, we calculated the proportion mediated as the ratio of the ACME to the TE. The mediation analysis was conducted using the R package “mediation”.^15^

# Figure S1. Histogram of neuroticism scores


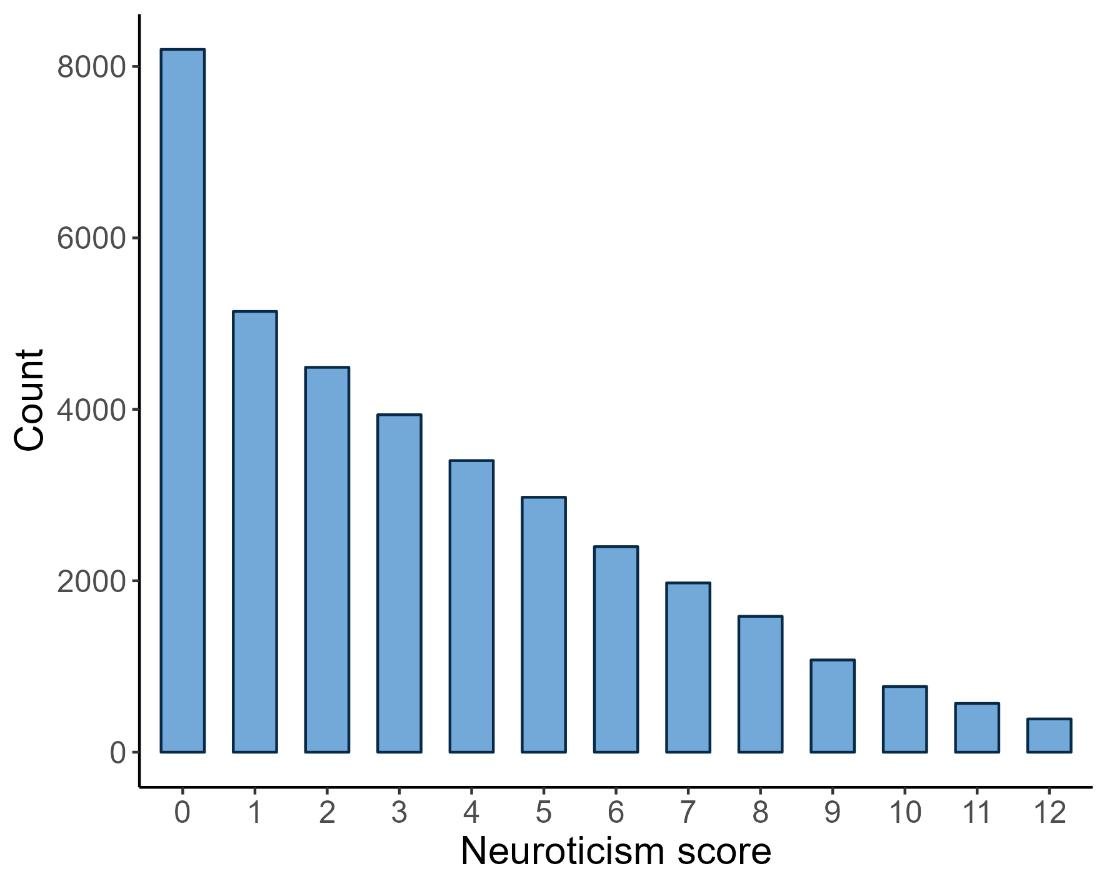


# Figure S2. Association between neuroticism and regional cortical volumes


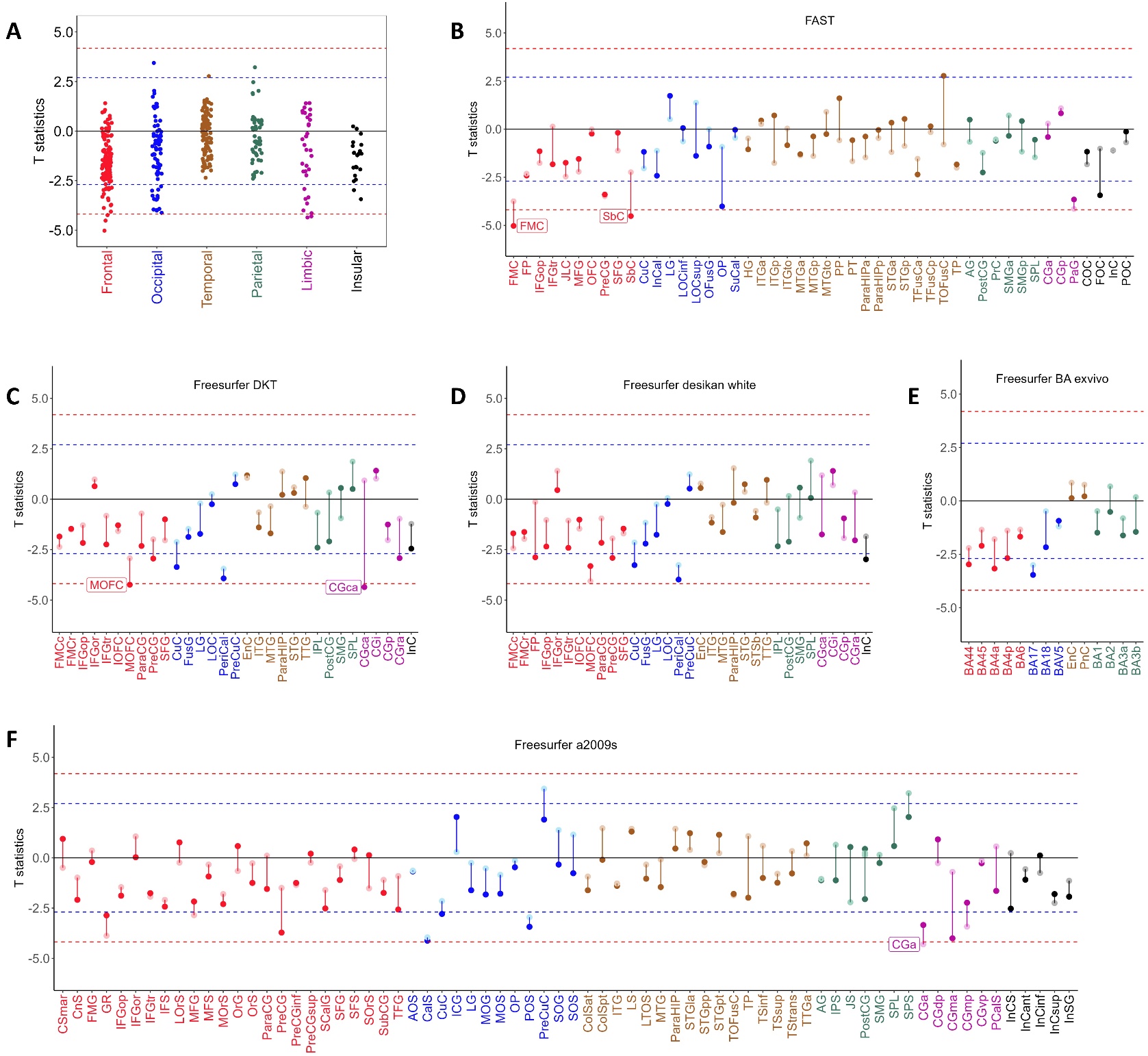


T statistics from the linear regression analysis of neuroticism with all imaging-derived phenotypes (IDPs) for regional cortical volumes (A), IDPs derived from FMRIB’s Automated Segmentation Tool (FAST) (B), and IDPs derived by FreeSurfer using different brain atlases: DKT (C), Desikan white (D), BA exvivo (E), and a2009s (F). Colours indicate different brain regions: red for the frontal lobe, blue for the occipital lobe, brown for the temporal lobe, green for the parietal lobe, purple for the limbic lobe, and black for the insular region. Darker points represent associations in the left hemisphere, and lighter points indicate the right hemisphere. Red dashed line indicates the Bonferroni threshold (1747 tests, p = 2.86×10^-5^, T statistics = ±4.18) and blue dashed line indicated the False Discovery rate threshold (1747 tests, p = 0.007, T statistics = ±2.70). See Table S5 for full list of abbreviations, regression coefficients, and 95% confidence intervals. FMC, Frontal Medial Cortex; SbC, Subcallosal cortex; MOFC, Medial Orbitofrontal Cortex; CGca, Cingulate Gyrus, caudal anterior; CGa, Cingulate gyrus, anterior.

# Figure S3. Association between neuroticism and regional cortical area


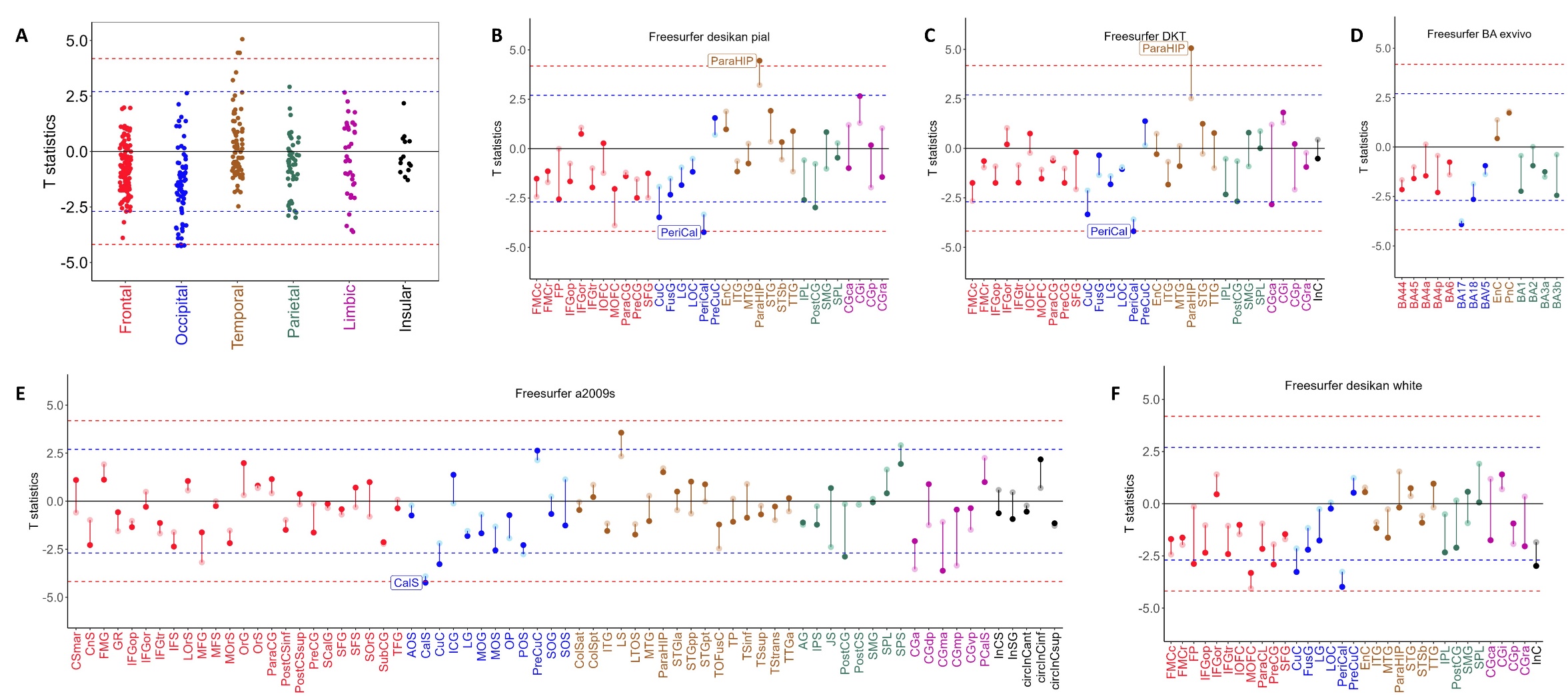


T statistics from the linear regression analysis of neuroticism with all imaging-derived phenotypes (IDPs) for regional cortical area (A) and IDPs derived by FreeSurfer using different brain atlases: Desikan pial (B), DKT (C), BA exvivo (D), a2009s (E), and Desikan white (F). Colours indicate different brain regions: red for the frontal lobe, blue for the occipital lobe, brown for the temporal lobe, green for the parietal lobe, purple for the limbic lobe, and black for the insular region. Darker points represent associations in the left hemisphere, and lighter points indicate the right hemisphere. Red dashed line indicates the Bonferroni threshold (1747 tests, p = 2.86*10^-5^, T statistics = ±4.18) and blue dashed line indicated the False Discovery rate threshold (1747 tests, p = 0.007, T statistics = ±2.70). See Table S5 for full list of abbreviations, regression coefficients, and 95% confidence intervals. PeriCal, Pericalcarine Cortex; ParaHIP, Parahippocampal gyrus; CalS, Calcarine Sulcus.

# Figure S4. Association between neuroticism and regional cortical thickness


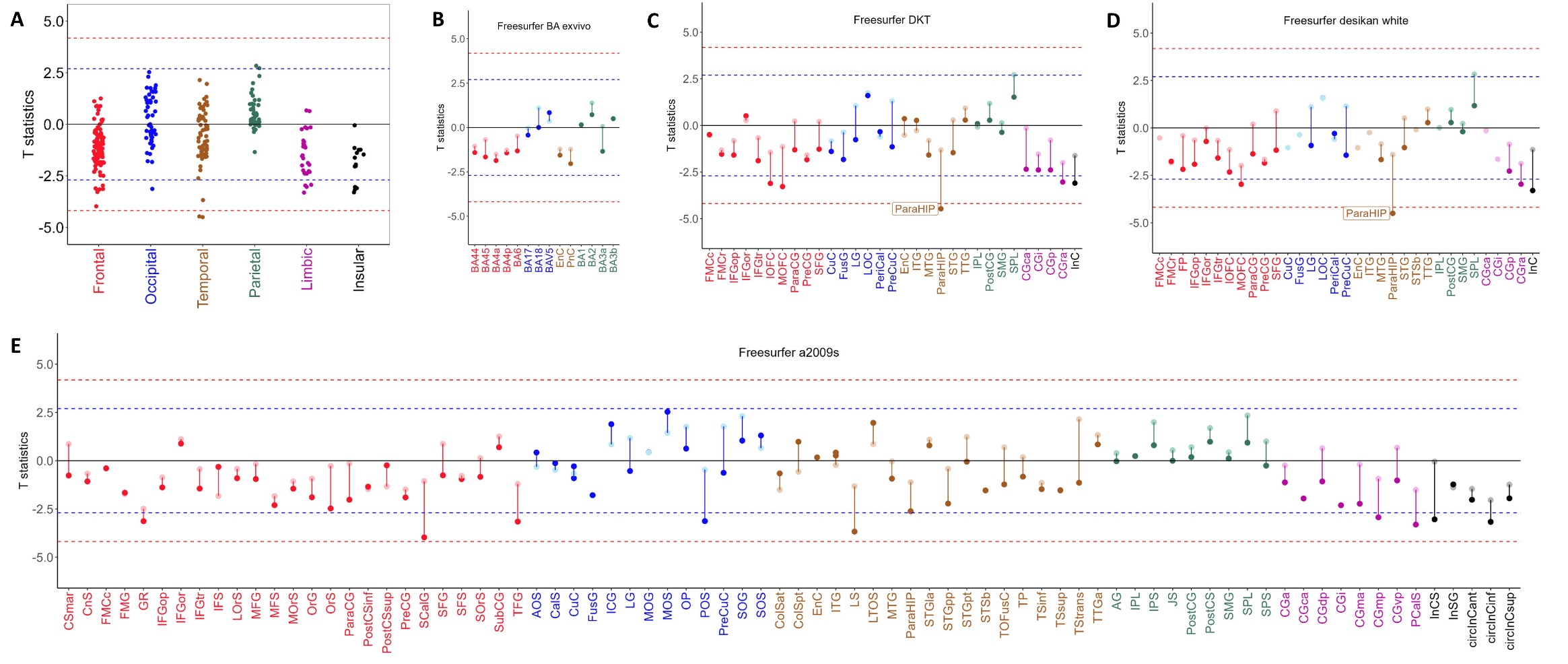


T statistics from the linear regression analysis of neuroticism with all imaging-derived phenotypes (IDPs) for regional cortical thickness (A) and IDPs derived by FreeSurfer using different brain atlases: BA exvivo (B), DKT (C), Desikan white (D), a2009s (E). Colours indicate different brain regions: red for the frontal lobe, blue for the occipital lobe, brown for the temporal lobe, green for the parietal lobe, purple for the limbic lobe, and black for the insular region. Darker points represent associations in the left hemisphere, and lighter points indicate the right hemisphere. Red dashed line indicates the Bonferroni threshold (1747 tests, p = 2.86*10^-5^, T statistics = ±4.18) and blue dashed line indicated the False Discovery rate threshold (1747 tests, p = 0.007, T statistics = ±2.70). See Table S5 for full list of abbreviations, regression coefficients, and 95% confidence intervals. ParaHIP, Parahippocampal gyrus.

# Figure S5. Association between neuroticism and regional grey-white matter contrast


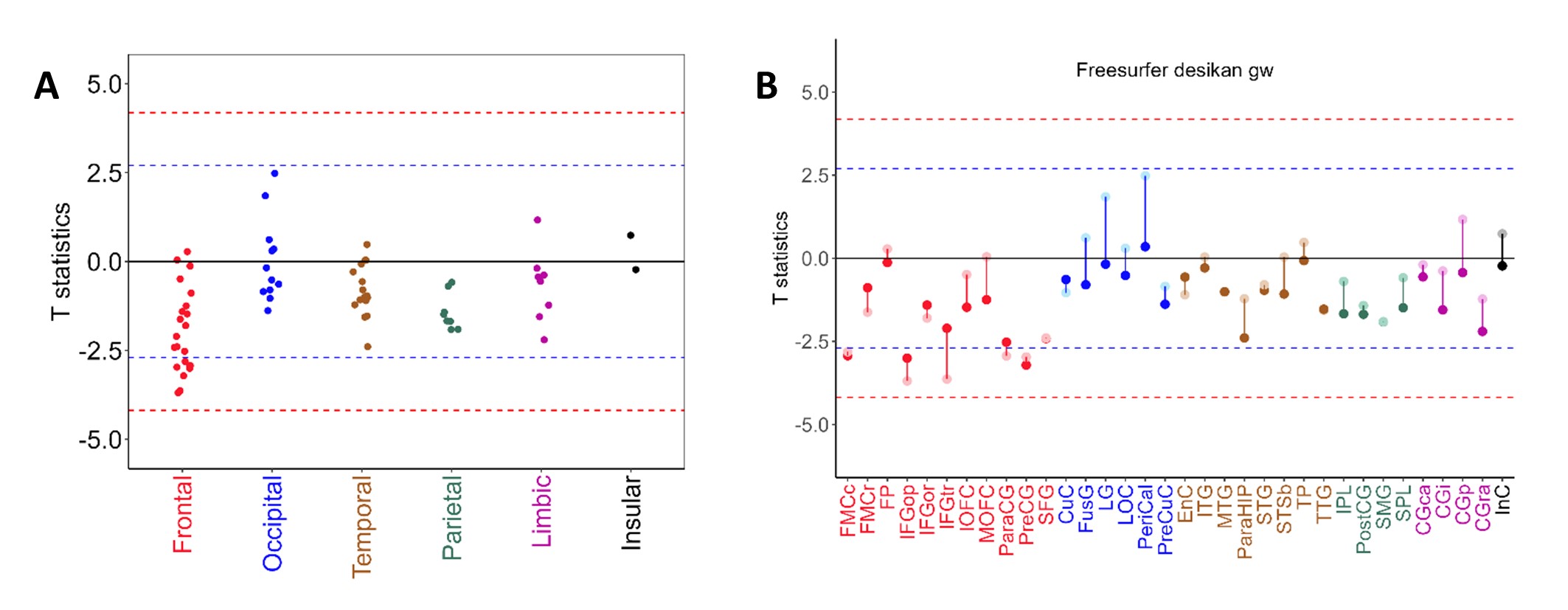


T statistics from the linear regression analysis of neuroticism with all imaging-derived phenotypes (IDPs) for regional grey-white matter contrast (A) and IDPs derived by FreeSurfer using desikan gw atlas (B). Colours indicate different brain regions: red for the frontal lobe, blue for the occipital lobe, brown for the temporal lobe, green for the parietal lobe, purple for the limbic lobe, and black for the insular region. Darker points represent associations in the left hemisphere, and lighter points indicate the right hemisphere. Red dashed line indicates the Bonferroni threshold (1747 tests, p = 2.86*10^-5^, T statistics = ±4.18) and blue dashed line indicated the False Discovery rate threshold (1747 tests, p = 0.007, T statistics = ±2.70). See Table S5 for full list of abbreviations, regression coefficients, and 95% confidence intervals.

# Figure S6. Association between neuroticism and subcortical volumes


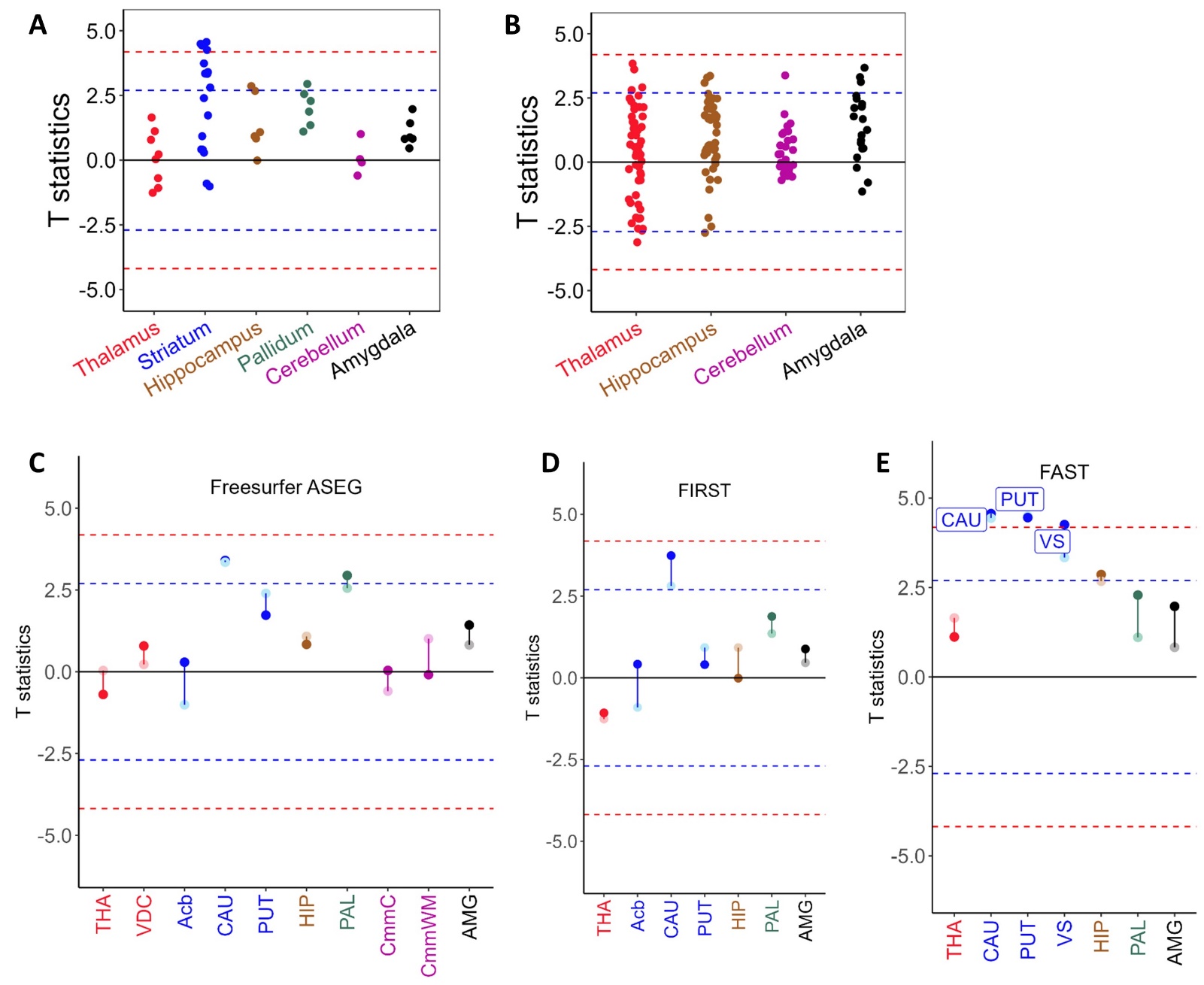


T statistics from the linear regression analysis of neuroticism with all imaging-derived phenotypes (IDPs) for subcortical volumes (A), IDPs derived by FreeSurfer subsegmentation (B) and aseg atlas (C), and IDPs derived from FMRIB’s Automated Segmentation Tool (FAST) (D), and FMRIB’s Integrated Registration and Segmentation Tool (FIRST) (E). Colours indicate different brain regions: red for thalamus, blue for striatum, brown for hippocampus, green for pallidum, purple for cerebellum, and black for amygdala. Darker points represent associations in the left hemisphere, and lighter points indicate the right hemisphere. Red dashed line indicates the Bonferroni threshold (1747 tests, p = 2.86*10^-5^, T statistics = ±4.18) and blue dashed line indicated the False Discovery rate threshold (1747 tests, p = 0.007, T statistics = ±2.70). See Table S5 for full list of abbreviations, regression coefficients, and 95% confidence intervals. CAU, Caudate; PUT, Putamen; VS, Ventral striatum.

# Figure S7. Association between neuroticism and white matter microstructure across tracts


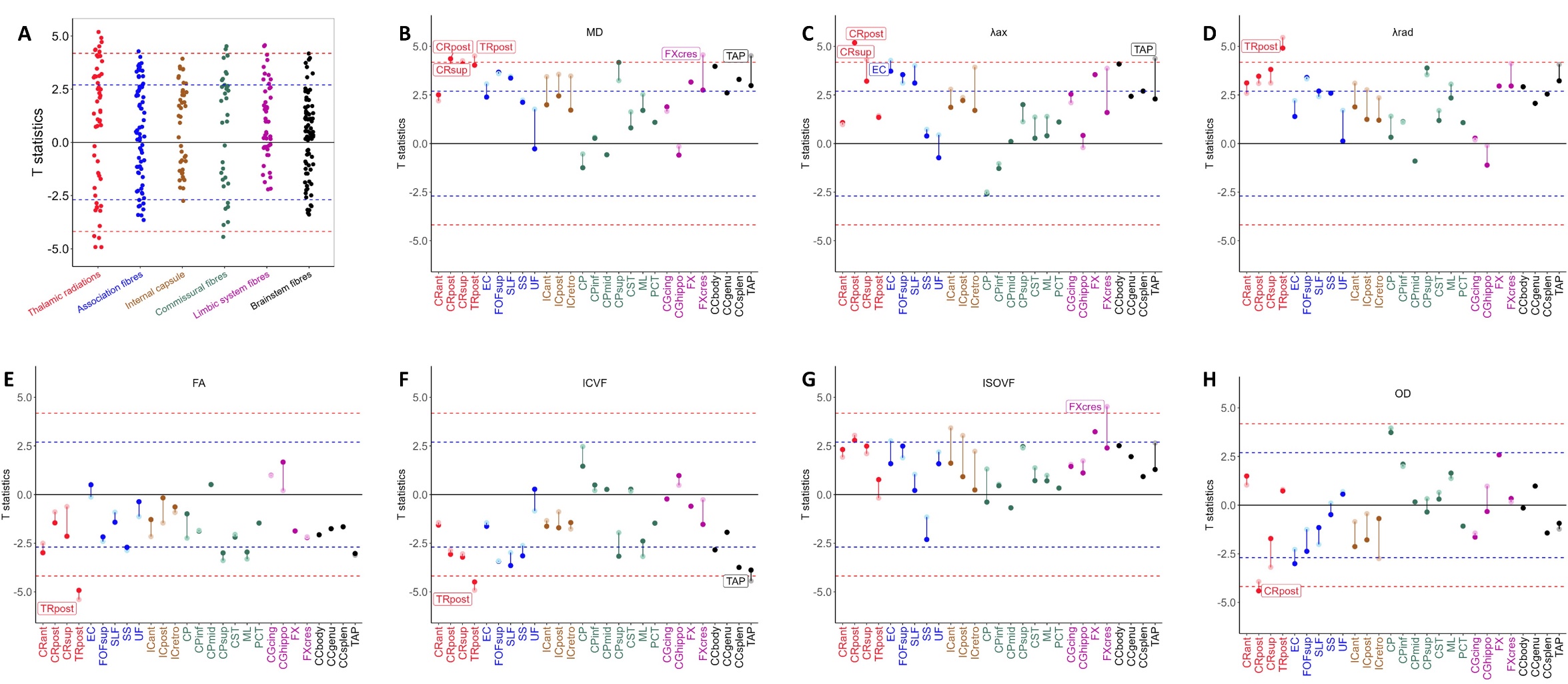


T statistics from the linear regression analysis of neuroticism with all imaging-derived phenotypes (IDPs) for white matter microstructure (A). Panels B–H show specific IDPs for mean diffusivity (MD), axial diffusivity (λax), radial diffusivity (λrad), fractional anisotropy (FA), intra-cellular volume fraction (ICVF), isotropic volume fraction (ISOVF), and orientation dispersion (OD), respectively. Colours indicate groups of tracts: red for thalamic radiations, blue for association fibres, brown for the internal capsule, green for commissural fibres, purple for limbic system fibres, and black for brainstem fibres. Darker points represent associations in the left hemisphere, and lighter points indicate the right hemisphere. Red dashed line indicates the Bonferroni threshold (1747 tests, p = 2.86×10^-5^, T statistics = ±4.18) and blue dashed line indicated the False Discovery rate threshold (1747 tests, p = 0.007, T statistics = ±2.70). See Table S5 for full list of abbreviations, regression coefficients, and 95% confidence intervals. TAP, tapetum; FXcres, fornix (cres)/stria terminalis; CRsup, superior corona radiate; CRpost, posterior corona radiate; TRpost, posterior thalamic radiation; EC, external capsule.

# Figure S8. Scatter plots and funnel plots of Mendelian randomization analyses for the association between neuroticism and IDPs with significant inverse variance weighted estimates


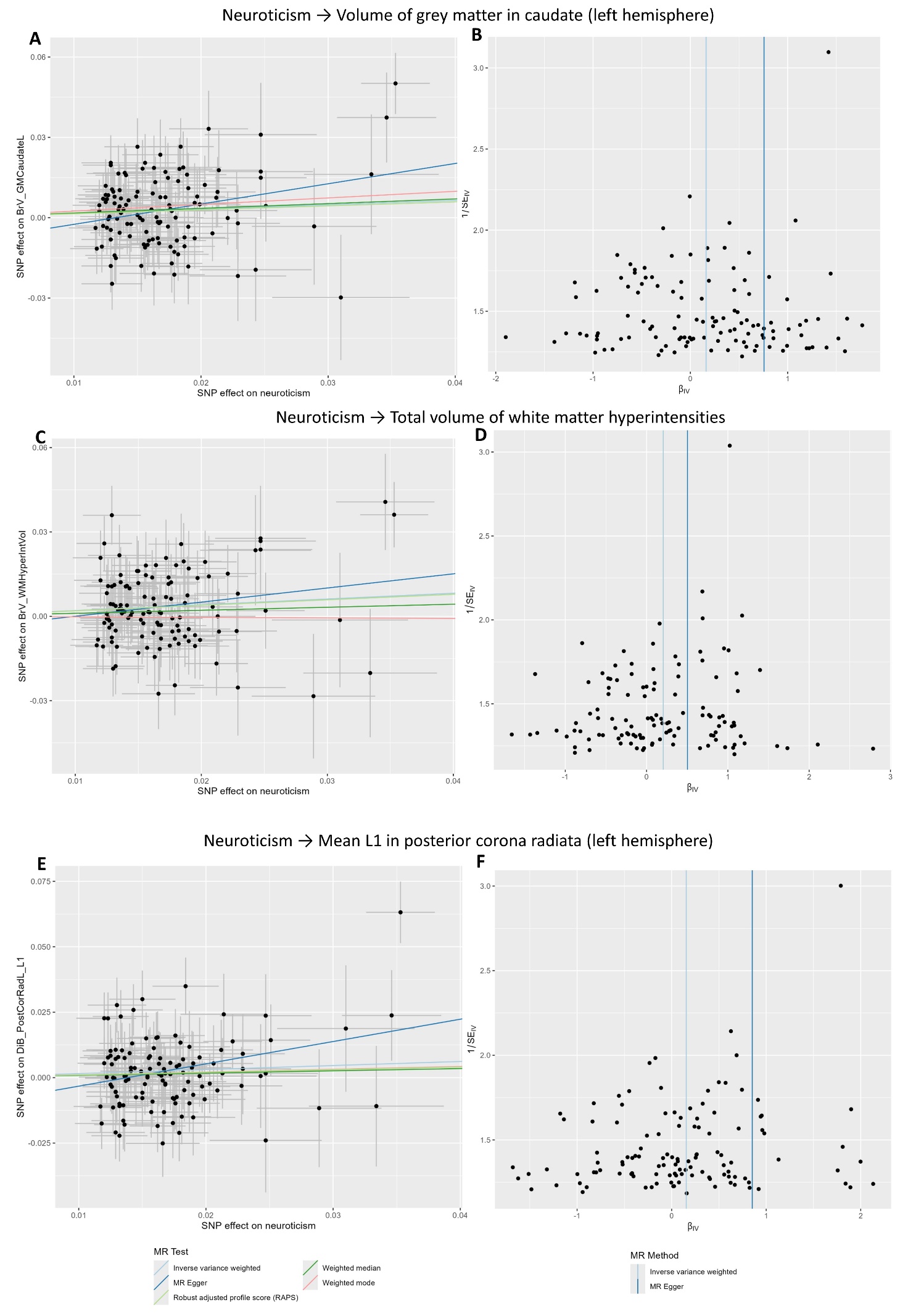


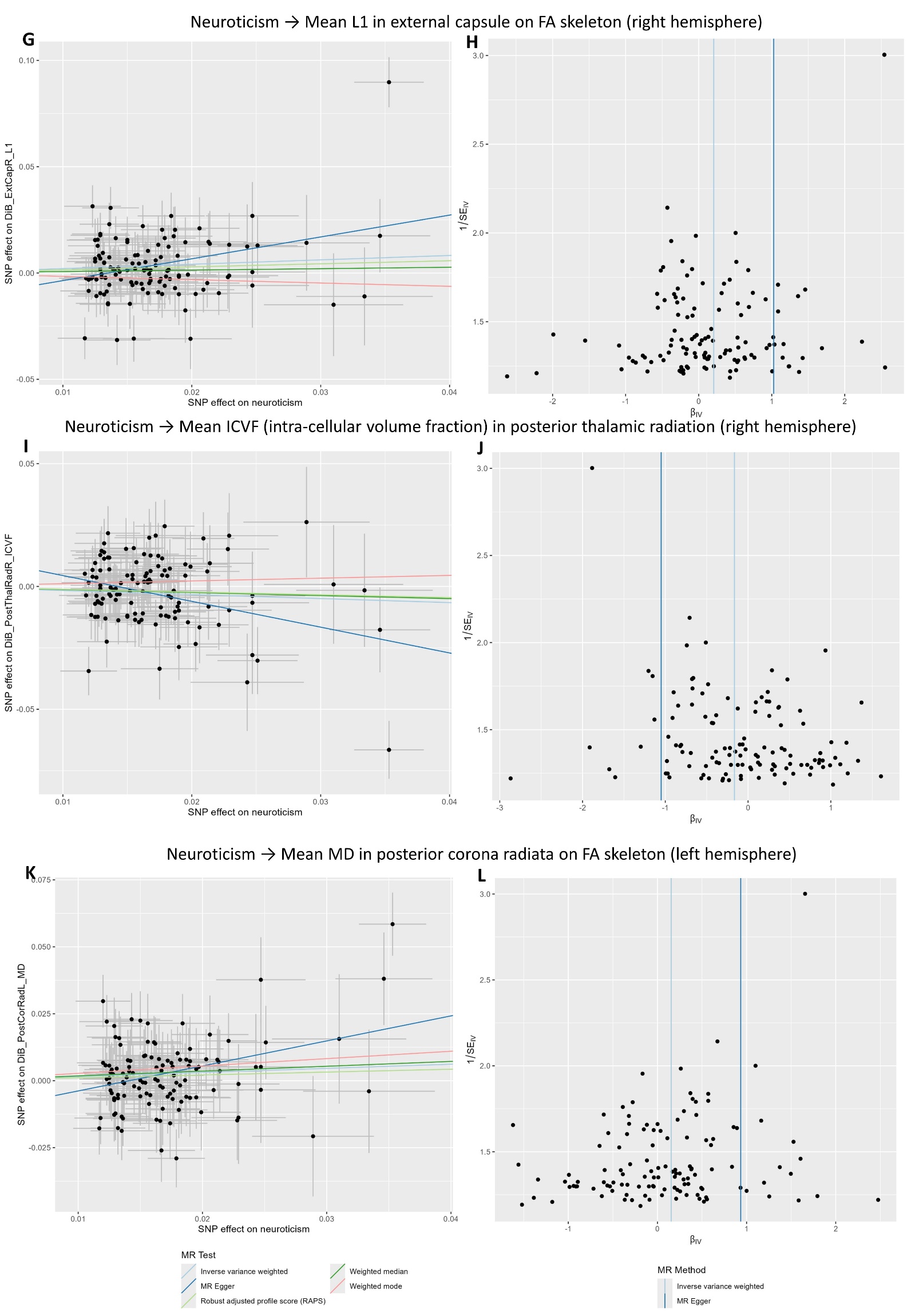


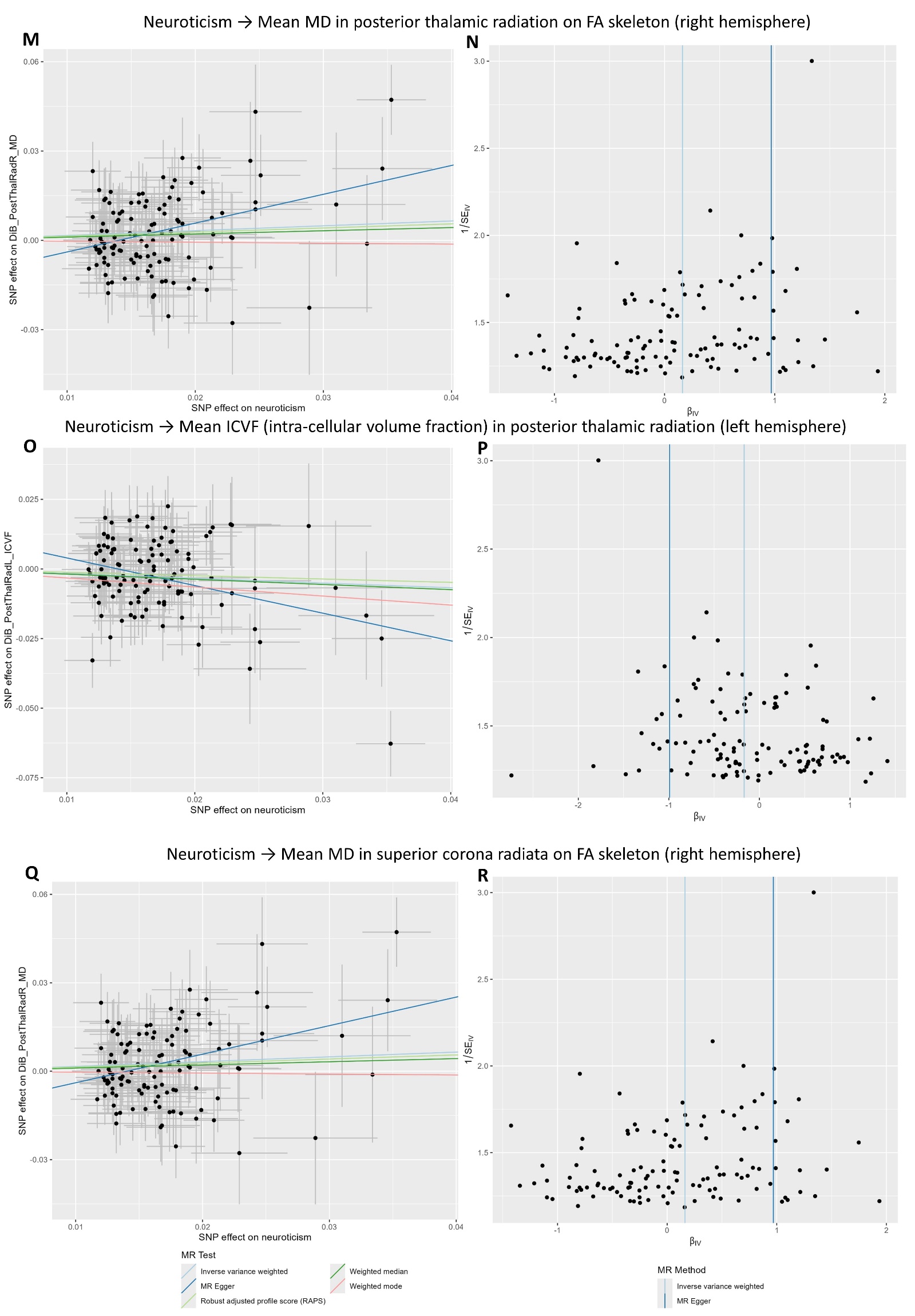


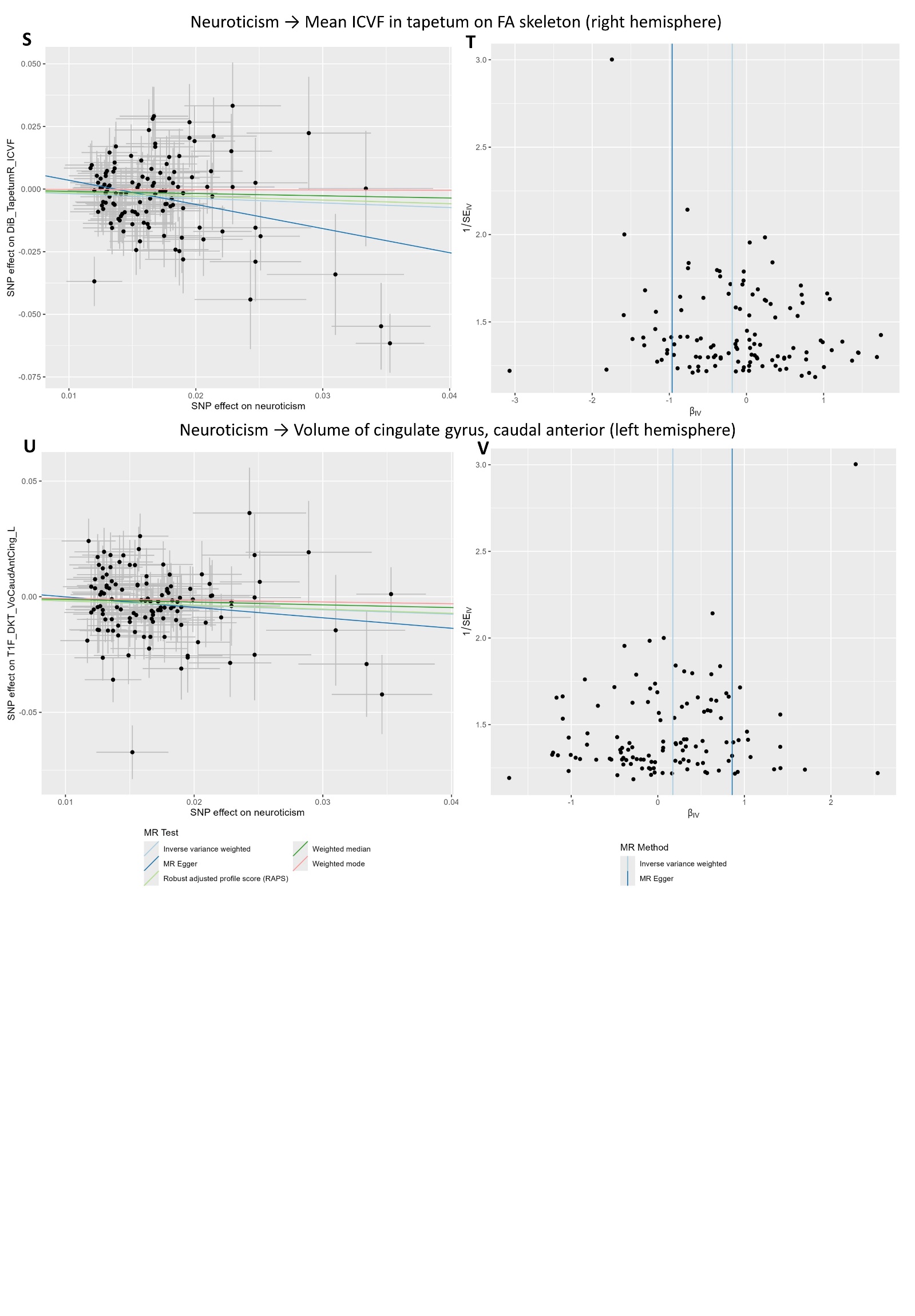


The scatter plots (A, C, E, G, I, K, M, O, Q, S, U) illustrate the associations between individual SNPs and both exposure and outcome variables, with dotted cross-hairs representing standard errors. Colored lines depict effect estimates derived from various MR methods. The funnel plots (B, D, F, H, J, L, N, P, R, T, V) display single-SNP effect estimates against their inverse standard errors.

# Figure S9. Scatter plots and funnel plots of Mendelian randomization analyses for the association between IDPs and neuroticism with significant inverse variance weighted estimates


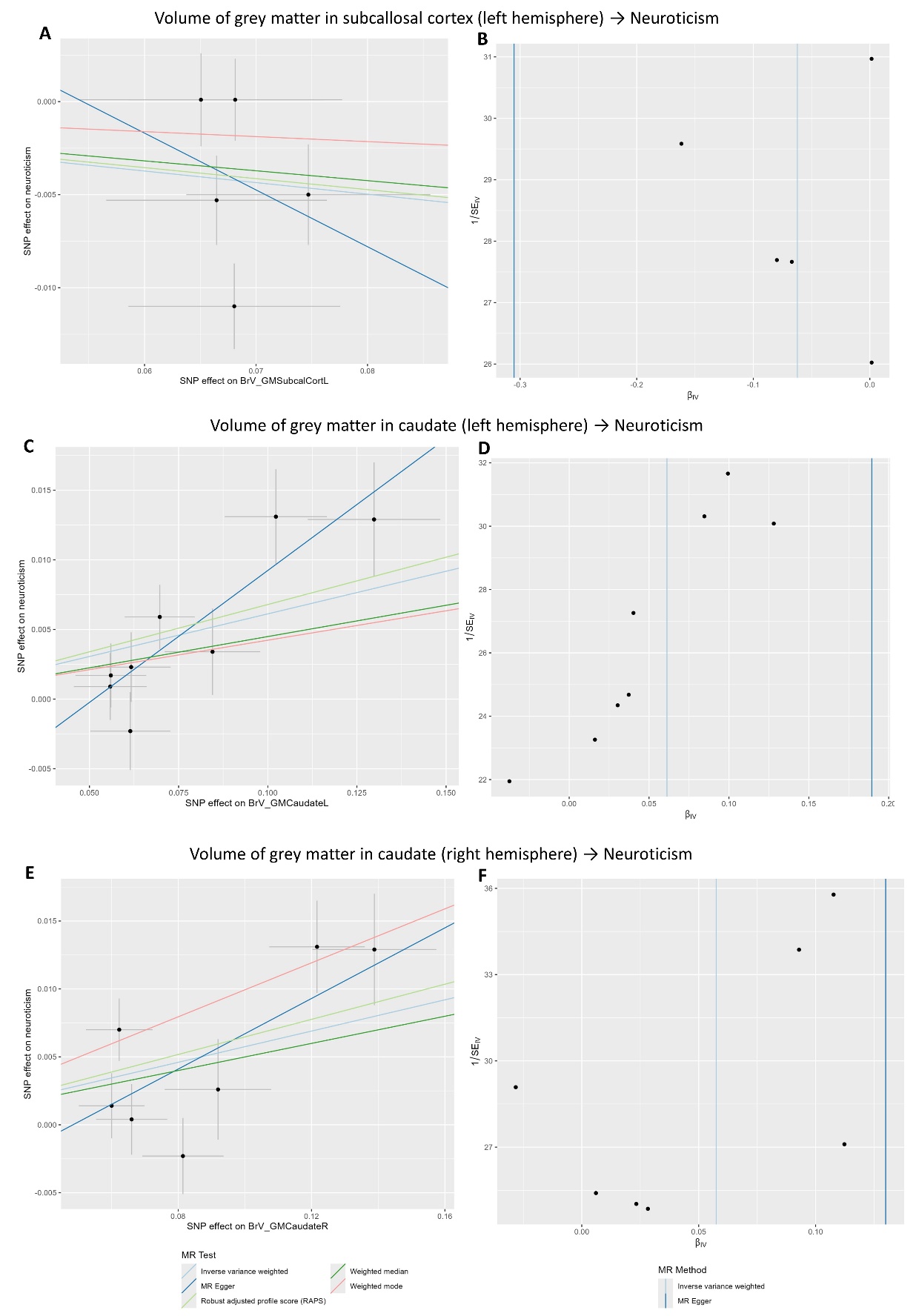


The scatter plots (A, C, E) illustrate the associations between individual SNPs and both exposure and outcome variables, with dotted cross-hairs representing standard errors. Colored lines depict effect estimates derived from various MR methods. The funnel plots (B, D, F) display single-SNP effect estimates against their inverse standard errors.

# Reference

1. Zhang Y, Brady M, Smith S. Segmentation of brain MR images through a hidden Markov random field model and the expectation-maximization algorithm. *IEEE Trans Med Imaging*. Jan 2001;20(1):45-57. doi:10.1109/42.906424

2. Patenaude B, Smith SM, Kennedy DN, Jenkinson M. A Bayesian model of shape and appearance for subcortical brain segmentation. *Neuroimage*. Jun 1 2011;56(3):907-22. doi:10.1016/j.neuroimage.2011.02.046

3. Zhang H, Schneider T, Wheeler-Kingshott CA, Alexander DC. NODDI: practical in vivo neurite orientation dispersion and density imaging of the human brain. *Neuroimage*. Jul 16 2012;61(4):1000-16. doi:10.1016/j.neuroimage.2012.03.072

4. Bowden J, Davey Smith G, Burgess S. Mendelian randomization with invalid instruments: effect estimation and bias detection through Egger regression. *International Journal of Epidemiology*. 2015;44(2):512-525. doi:10.1093/ije/dyv080

5. Bowden J, Davey Smith G, Haycock PC, Burgess S. Consistent Estimation in Mendelian Randomization with Some Invalid Instruments Using a Weighted Median Estimator. *Genet Epidemiol*. May 2016;40(4):304-14. doi:10.1002/gepi.21965

6. Hartwig FP, Davey Smith G, Bowden J. Robust inference in summary data Mendelian randomization via the zero modal pleiotropy assumption. *International Journal of Epidemiology*. 2017;46(6):1985-1998. doi:10.1093/ije/dyx102

7. Qingyuan Z, Jingshu W, Gibran H, Jack B, Dylan SS. Statistical inference in two-sample summary-data Mendelian randomization using robust adjusted profile score. *The Annals of Statistics*. 6/1 2020;48(3):1742-1769. doi:10.1214/19-AOS1866

8. Verbanck M, Chen CY, Neale B, Do R. Detection of widespread horizontal pleiotropy in causal relationships inferred from Mendelian randomization between complex traits and diseases. *Nat Genet*. May 2018;50(5):693-698. doi:10.1038/s41588-018-0099-7

9. Mounier N, Kutalik Z. Bias correction for inverse variance weighting Mendelian randomization. *Genet Epidemiol*. Jun 2023;47(4):314-331. doi:10.1002/gepi.22522

10. Hemani G, Tilling K, Davey Smith G. Orienting the causal relationship between imprecisely measured traits using GWAS summary data. *PLoS Genet*. Nov 2017;13(11):e1007081. doi:10.1371/journal.pgen.1007081

11. Bowden J, Davey Smith G, Burgess S. Mendelian randomization with invalid instruments: effect estimation and bias detection through Egger regression. *Int J Epidemiol*. Apr 2015;44(2):512-25. doi:10.1093/ije/dyv080

12. Burgess S. Sample size and power calculations in Mendelian randomization with a single instrumental variable and a binary outcome. *Int J Epidemiol*. Jun 2014;43(3):922-9. doi:10.1093/ije/dyu005

13. Smith SM, Douaud G, Chen W, et al. An expanded set of genome-wide association studies of brain imaging phenotypes in UK Biobank. *Nat Neurosci*. May 2021;24(5):737-745. doi:10.1038/s41593-021-00826-4

14. Kosuke I, Luke K, Teppei Y. Identification, Inference and Sensitivity Analysis for Causal Mediation Effects. *Statistical Science*. 2/1 2010;25(1):51-71. doi:10.1214/10-STS321

15. Tingley D, Yamamoto T, Hirose K, Keele L, Imai K. Mediation: R package for causal mediation analysis. 2014;
