# Supplementary material for: Association between neuroticism and brain-wide structural outcomes: Mediation by vascular and mental conditions
Source: Psychol Med. Author manuscript; Available in PMC 2026 Feb 18. (PMC7618729; doi:10.1017/S0033291725102390)
Supplement: Supplementary file 1 [file EMS212281-supplement-Supplementary_Information.pdf]

Supplementary material. The supplementary material for this article can be found at http://doi.org/10.1017/S0033291725102390.

https://doi.org/10.1017/S0033291725102390 Published online by Cambridge University Press
